# The Aryl Hydrocarbon Receptor (AhR) Mediates the Counter-Regulatory Effects of Pelargonidins in Models of Inflammation and Metabolic Dysfunctions

**DOI:** 10.3390/nu11081820

**Published:** 2019-08-07

**Authors:** Michele Biagioli, Adriana Carino, Chiara Fiorucci, Giannamaria Annunziato, Silvia Marchianò, Martina Bordoni, Rosalinda Roselli, Cristina Di Giorgio, Federica Castiglione, Patrizia Ricci, Agostino Bruno, Andrea Faccini, Eleonora Distrutti, Monia Baldoni, Gabriele Costantino, Stefano Fiorucci

**Affiliations:** 1 Dipartimento di Scienze Biomediche e Chirurgiche, Università di Perugia, 06100 Perugia, Italy; 2 Department of Food and Drugs, University of Parma, 43100 Parma, Italy; 3 Department of Pharmacy, University of Naples Federico II, 80100 Naples, Italy; 4 Centro Interdipartimentale Misure ‘G. Casnati’—University of Parma, 43100 Parma, Italy; 5 Azienda Ospedaliera di Perugia, 06100 Perugia, Italy

**Keywords:** pelargonidins, aryl hydrocarbon receptor, colitis, hepatic steatosis, inflammation

## Abstract

Pelargonidins are anthocyanidins thought to be beneficial for the human health, although controversies exist over the doses needed and the unclear mechanism of action, along with poor systemic bioavailability. One putative target of pelargonidins is the aryl hydrocarbon receptor (AhR). A synthetic pelargonidin (Mt-P) was synthesized by the methylation of the pelargonidin (the natural compound indicated as P). Mt-P transactivated the AhR with an EC_50_ of 1.97 µM and was ~2-fold more potent than the natural compound. In vitro Mt-P attenuated pro-inflammatory activities of Raw264.7 macrophage cells in an AhR-dependent manner. In vivo, administration of the Mt-P in Balb/c mice resulted in a dose-dependent attenuation of signs and symptoms of colitis induced by TNBS. A dose of 5 mg/kg Mt-P, but not the natural compound P, reversed intestinal inflammation and increased expression of Tnf-α, Ifn-ƴ, and Il-6, while promoted the expansion of regulatory T cells and M2 macrophages. In C57BL/6J mice fed a high fat diet (HFD), Mt-P attenuated body weight gain, intestinal and liver inflammation, and ameliorated insulin sensitivity, while worsened liver steatosis by up-regulating the liver expression of Cd36 and Apo100b. These effects were abrogated by AhR gene ablation. Mt-P is a synthetic pelargonidin endowed with robust AhR agonist activity that exerts beneficial effects in murine models of inflammation and metabolic dysfunction.

## 1. Introduction

The aryl hydrocarbon receptor (AhR) is a ligand-activated nuclear receptor that belongs to the basic helix-loop-helix (bHLH)/PerARNT-Sim (PAS) superfamily. Although AhR was originally described as a xenobiotics sensor, it is now well established that dietary/intestinal microbiota metabolites such as kynurenines represent the physiological ligands [1]. Similarly to other nuclear receptors, in the absence of a ligand, AhR resides in cytoplasm as a component of a chaperone complex that includes a dimer of heat shock protein 90 (Hsp90), together with the cochaperones such as p23, AhR interacting protein (AIP), and the immunophilin-like AH receptor-interacting protein that binds to and inactivates the receptor [2]. This multiprotein complex is released upon agonist ligation, allowing the receptor to translocate to the nucleus to form a heterodimeric complex with the AhR nuclear translocator (ARNT). The AhR/ARNT dimer is then recruited to the promoter of AhR target genes, where it binds to a consensus sequence known as dioxin or xenobiotic response elements (XREs), allowing the transcription of a large family of AhR-dependent proteins including xenobiotic metabolizing enzymes [3]. 

The AhR is widely expressed by cells of innate/adaptive immunity [4]. In the gastrointestinal tract, several immune cell subsets—including T helper (Th) 17 cells, IL-22 secreting group 3 innate lymphoid cells (ILC3s), and regulatory T cells (Treg)—express the receptor, providing a network of regulatory signals essential for development and maintenance of immune tolerance [5,6]. Accordingly, not only is AhR gene expression negatively regulated in patients with inflammatory bowel disease (IBD) [7], but AhR ligands have been proven effective in attenuating inflammation and immune dysfunction in murine models of colitis, while AhR^−/−^ mice are more prone than their wild-type counterparts to develop severe immune dysfunction [8,9,10,11]. 

In addition to immune cells, high levels of AhR are detected in intestinal epithelial cells and the receptor appears essential for the maintenance of the intestinal barrier integrity [1]. Since a compromised integrity of the intestinal barrier has substantial implications for human health even beyond the intestine, AhR has emerged as potential target in the treatment of highly prevalent human disorders.

Chronic exposure to a high caloric intake resulting in metabolic syndrome, diabetes, and obesity compromises the integrity of intestinal barrier function, promoting leakage of bacterial products, a state of subclinical inflammation, and accumulation of inflammatory mediators in metabolic tissues such as the white adipose tissue (WAT) and liver [12,13]. Accordingly, changes in intestinal barrier function, promoted by dysbiotic intestinal microbiota and/or genetic and dietetic factors, promote transition from the simple steatosis to steato-hepatitis, representing a validated target in the treatment of non-alcoholic steato-hepatitis (NASH), a highly prevalent human disease for which therapy is still sub-optimal [14].

Anthocyanins are a subgroup of flavonoids derived from the respective aglycons (anthocyanidins), from which they are differentiated by the addition of a glycosidic group [15,16]. Pelargonidin is a water soluble anthocyanidin responsible for the red/orange colors of berries such as ripe raspberries and strawberries, as well as blueberries, blackberries, cranberries, but also saskatoon berries and chokeberries too. It is also found in plums and pomegranates, confers red radishes their color, and occurs in nature as glycosylated derivatives [17]. While, similarly to other anthocyanins, pelargonidin is thought to be beneficial for human health [18,19,20], controversies exist over the doses needed to reach these effects, along with poor systemic bioavailability and unclear mechanism(s) of action. 

In the present study, we report the generation and pharmacological characterization of a synthetic pelargonidin, obtained by de novo synthesis, endowed with robust AhR agonist activity. By using pharmacological and genetic approaches, we demonstrate that the novel derivative activates the AhR in the intestine and metabolic tissues, and is beneficial in reducing inflammation in murine models of intestinal inflammation and NASH. 

## 2. Materials and Methods

### 2.1. Chemistry

All reagents were purchased from Sigma-Aldrich (St. Louis, MO, USA), Alfa Aesar (Haverhill, MA, USA) and Enamine (Monmouth Jct, NJ, USA) at reagent purity and, unless otherwise noted, used without any further purification. Dry solvents used in the reactions were obtained by distillation of technical grade materials over appropriate dehydrating agents. Reactions were monitored by thin layer chromatography on silica gel-coated aluminum foils (silica gel on Al foils, SUPELCO Analytical, Sigma-Aldrich) at 254 and 365 nm. Where indicated, intermediates and final products were purified by silica gel flash chromatography (silica gel, C18 reverse phase), using appropriate solvent mixtures. ^1^H NMR and ^13^C NMR spectra were recorded on a BRUKER AVANCE spectrometer at 400 and 100 MHz, respectively, with TMS as internal standard. All compounds were tested at purity of 95% or higher (by HPLC/MS).

### 2.2. Extraction and Hydrolysis of Natural Pelargonidins 

The edible red radish (European variety) (1 kg) was purchased from a local supermarket, Parma, Italy. It was stored at 4 °C and washed thoroughly prior to use. Washed red radish was peeled into small pieces prior to solvent extraction. Pelargonidins were extracted by stirring 5 g of finely-cut peel with 20 mL of methanol for 18 h at room temperature. The extract was then filtered on a paper filter. After filtration on a 0.2 µm PTFE filter, the extract was purified by silica gel in reverse phase starting from H_2_O/MeOH/HCOOH (9:1:0.01) to MeOH 100%, and the anthocyanins fraction was collected and dried under N_2_ flux. The pelargonidins purified extract (300 mg) was dissolved in HCl 1N (10 mL) and allowed to react at 100 °C for 3 h. After filtration on a 0.2 µm PTFE filter, the reaction mixture was dried under N_2_ flux and purified by silica gel on reverse phase, starting from H_2_O/MeOH/HCOOH (9:1:0.01) to MeOH 100%, and the pelargonidins fraction collected and dried under N_2_ flux [21]. HRMS (ESI) calculated for C_15_H_11_O_5_ ([M + H]^+^) 272.0606; found 272.0600.

### 2.3. Synthesis of Methylated Pelargonidin

**Scheme 1:**
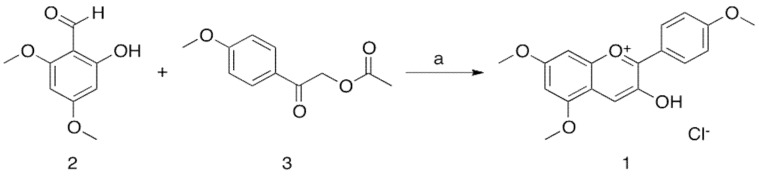
Methylated pelargonidin (Mt-P) synthesis.

For the synthesis of methylated pelargonidin (Mt-P), the starting materials 2 and 3 were commercially available. Compounds 2 (350 mg, 1 eq) and 3 (336 mg, 1.1 eq) were dissolved in AcOEt/MeOH (1:2.5 mL) and gaseous HCl was bubbled for 1 h in the mixture at room temperature. The solution became dark red. After 1 h, the reaction was allowed to react at room temperature overnight. After this period Et_2_O was added to the reaction mixture; after 40 min, the crude was filtered by a Buckner funnel and the red powder collected and triturated with Et_2_O with 0.1% of formic acid. Finally, the product was filtered with a Buckner funnel and collected (Scheme 1) [22]. 

Purified by filtration and washed with Et2O. Yield: 33% (brown-red powder); ^1^H-NMR (DMSO 300 MHz): δ 2.74 (s, 3H), 3.01 (s, 3H), 3.84 (s, 3H), 6.69 (s, 1H), 6.91 (s, 1H), 7.18 (d, *J* = 9.25, 2H), 8.60 (d, *J* = 9.25, 2H), 8.55 (s, 1H), 13.02 (s, 1H). HRMS (ESI) calculated for C_18_H_17_O_5_ ([M + H]^+^) 314.3246; found 314.3200. 

### 2.4. LC-HRMS Analyses

Liquid Chromatography—High Resolution Mass (LC–HRMS) analyses were carried out on a DIONEX Ultimate3000 Thermo Fisher Scientific equipped with a Ultimate300 Variable Wavelength Detector and a LTQ Orbitrap XL (Thermo Fisher Scientific; Waltham, MA, USA). Electrospray ionization at positive ion modes was performed with a spray voltage 3 kV; capillary voltage 13 V; source temperature 275 °C; tube lens 80 V; sheath gas flow rate 20; aux gas flow rate 5; and sweep gas flow rate 5. The mass data acquisition was performed by two scan event: Scan event 1, FTMS + *p* res = 30,000 o (200–1500); scan event 2, FTMS + *p* res = 30,000 o (200–1500) SID = 35 V.

Samples were injected on an Aeris Peptide 3.6u XB-C18 2.1 mm × 15 cm (Phenomenex) The mobile phase consisted of water with 0.1% formic acid (solvent A) and methanol with 0.1% formic acid (solvent B). Gradient: 0–5 min 95% A; 5–45 min from 95% A to 5% A; 45–51 min 5% A; 51–52 from 5% to 95% A; 52–60 95% A. Flow rate was 0.2 ml/min. UV/Vis analyzer wavelength: 280 nm, 440 nm, 508 nm, 522 nm. Column temperature 40 °C.

### 2.5. Homology Model of the PAS B Domain of hAhR and Docking Studies 

The X-ray crystal structure of the Hypoxia Inducing Factor (HIF)-1α PasB domain (pdb code 4H6J) was used as template to generate the 3D model structure of the AhR PAS B domain. Twenty 3D models were generated using Modeller software. The obtained models were scored using the available scoring functions in Modeller 9 software (DOPE score) and through visual inspections [23]. The protonation state of ionizable residues was fixed by using the Protein Preparation Wizard of the Maestro graphical user interface and then checked by visual inspection. The geometry of the final model was optimized using several cycles of OPLS3 force field (available in Maestro) minimization, until a gradient of 0.001 kcal/(mol·Å^2^) was reached. The structure thus obtained was used to perform docking studies by using Glide. The 3D structure of the ligand was built using the fragment builder tool of Maestro. The compound was geometrically optimized by means of Macromodel, using MMFFs as force field and water as implicit solvent until a convergence value of 0.05 kcal/(mol·Å^2^). The computational protocol applied consists of the application of 500 steps of the Polak−Ribieŕe conjugate gradient (PRCG) for structure minimizations. The grid was centered on the His291 and Ser365 residues. Each docking run was carried out with the standard precision (SP) method, and the van der Waals scaling factor of nonpolar atoms was set to 0.8. Twenty docking poses were obtained, and among these poses we selected the best pose in accordance to (i) the Glide scoring function and that (ii) able to interact at the same time with His291 and Ser365 residues. The selected docking pose was minimized using OPLS3 as force field and the PRCG method until a gradient of 0.001 kcal/(mol·Å^2^) was obtained, applying a stepwise relaxation protocol for which harmonic constraints were progressively reduced for backbone, side chains, and ligand atoms.

### 2.6. Cell Culture 

HepG2 cells, a human liver cancer cell line from ATCC (Manassas, VA, USA), were cultured at 37 °C in E-MEM supplemented with 10% FBS, 1% glutamine, and 1% penicillin/streptomycin. RAW264.7 cells, a murine macrophage cell line, were from ATCC (Manassas, VA, USA); EL4 cells, a murine T lymphocyte cell line, were from ATCC (Manassas, VA, USA); Caco-2 cells, a human intestinal epithelial cell line, were from Sigma-Aldrich (Saint Louis, MI, USA). Raw264.7, EL4, and Caco-2 cells were grown at 37 °C in D-MEM containing 10% FBS, 1% l-glutamine, and 1% penicillin/streptomycin. Cells were regularly passaged to maintain exponential growth. RAW264.7, EL4, and Caco-2 cells were classically activated with LPS (5 ng/mL, Sigma-Aldrich L2880) + IFN-γ (20 ng/mL, eBioscience, San Diego, CA, USA) for 16 h alone or in combination with P or Mt-P (0.1, 1, 10, and 20 µM) for the analysis of expression of several genes. 

### 2.7. Transactivation Assay

For AhR-mediated transactivation, HepG2 cells were plated at 5 × 10^4^ cells/well in a 24-well plate. Cells were transfected with 200 ng of the reporter vector pLightAhRE, 100 ng of pCMVXL4AhR, and 100 ng of pGL4.70 (Promega, Madison, WI, USA), a vector encoding the human Renilla luciferase gene. To evaluate FXR-mediated transactivation, HepG2 cells were transfected with 100 ng of human pSG5-FXR, 100 ng of human pSG5-RXR, 200 ng of the reporter vector p(hsp27)-TK-LUC containing the FXR response element IR1 cloned from the promoter of heat shock protein 27 (hsp27), and with 100 ng of pGL4.70 Renilla. To investigate the specificity of these compounds versus PXR, HepG2 cells were transfected with 100 ng pSG5-PXR, 100 ng pSG5-RXR, 100 ng pGL4.70 Renilla, and 200 ng reporter vector containing the PXR target gene promoter (CYP3A4 gene promoter) cloned upstream of the *luciferase* gene (pCYP3A4promoter-TKLuc). To evaluate LXRα-mediated transactivation, HepG2 cells were transfected with 20 ng of the reporter vector p(UAS)5XTKLuc, 100 ng of a vector containing the ligand binding domain of LXRα cloned upstream of the GAL4-DNA binding domain (i.e., pSG5-LXRαLBD-GAL4DBD), and 100 of pGL4.70 (Promega, Madison, WI, USA), a vector encoding the human Renilla gene. To evaluate GPBAR1-mediated transactivation, HEK-293T cells were transfected with 200 ng of human pGL4.29 (Promega, Madison, WI, USA), a reporter vector containing a cAMP response element (CRE) that drives the transcription of the luciferase reporter gene luc2P, with 100 ng of pCMVS.PORT6-human GPBAR1, and with 100 ng of pGL4.70 Renilla. At 24 h post-transfection, cells were stimulated 18 h with 5 nM TCDD, 10 μM CDCA, 500 nM Rifaximin, 10 μM GW3965, or 10 μM TLCA, and both pelargonidin and methylated pelargonidin (10 and 50 μM). Luciferase activities were assayed and normalized with Renilla activities. Dose-response curves were performed in HepG2 cells transfected with AhR, as described above, and then treated with increasing concentrations of both pelargonidin and methylated pelargonidin (1, 5, 10, 25, and 50 μM). At 18 h post-stimulation, cellular lysates were assayed for luciferase and Renilla activities using the Dual-Luciferase Reporter assay system (E1980, Promega, Madison, WI, USA). Luminescence was measured using Glomax 20/20 luminometer (Promega, Madison, WI, USA). Luciferase activities (RLU) were normalized with Renilla activities (RRU).

### 2.8. Wound-Healing Assay

To evaluate the intestinal mucosal healing, we used a classic in vitro wound healing assay, which consists in creating a “scratch” in a cell monolayer. Caco-2 cells were grown to confluence (3 days) on 12-well culture plates (1 × 10^5^ cells/well) and then starved overnight in 0.5% FBS containing medium. The cell monolayers were scraped in a straight line using a p200 sterile pipette tip in order to create a “scratch”; cell debris were removed by washing with PBS. The cells were then incubated with 2 mL of 0.5% FBS containing medium with or without LPS (1 µg/Ml) alone or in combination with P or Mt-P (20 µM). Cells treated only with the vehicle (NT) or only with TGFβ (5 ng/mL) were used as negative and positive control wells. To measure the migratory response of the cells into the scrape wounds, microscopic photographs were taken at 0 h and 24 h with a digital camera on an inverted microscope at 10× magnification. Images were analyzed by NIH ImageJ software (NIH, Bethesda, MD, USA) to calculate the area of scratch wound and represented as % of wound healed relative to the control wells (that was arbitrarily assigned as 100%). 

### 2.9. Chromatin Immunoprecipitation (ChIP) 

For ChIP assay, we studied the promoter of the murine gene encoding for the CYP1a1, investigating the sequence for the binding of AhR/ARNT dimer. RAW264.7 cells were exposed or not to LPS with P or Mt-P for 16 h and then crosslinked with formaldehyde. Chromatin immunoprecipitation (ChIP) assays were performed according the manufactured protocols (EZ-ChIP™ Upstate, #17-371, Millipore, Burlington, MA, USA). Briefly, cell extracts were sonicated and divided. Antibodies (5 µg) against AhR and normal goat IgG were added for immunoprecipitation. The immune-precipitated chromatin was recovered and purified. The *CYP1a1* promoter DNA was quantified by rtPCR analysis using primers around the binding site in the proximal promoter of *CYP1a1* (for GTGCAGATACCTCCCAGACC; rev AAGCAGTCCCTTCATGTGCT). Primers spanning a region located at ~14,000 kb downstream of the *CYP1a1* transcriptional start site (ACACAGCCCATTAGGATTCG; rev TGGGGAAAGAGGACACGTAG) were used as control. At least three replicates of each group were performed.

### 2.10. Animals 

Balb/c mice were purchased from Charles River (Wilmington, MA, USA). AhR null mice on C57BL/6 background and C57BL/6 congenic littermates were originally purchased from Charles River (Wilmington, MA, USA). The colonies were maintained in the animal facility of University of Perugia. Mice were housed under controlled temperatures (22 °C) and photoperiods (12:12-h light/dark cycle), allowed unrestricted access to standard mouse chow and tap water, and allowed to acclimatize to these conditions for at least 5 days before inclusion in an experiment. The study was conducted in agreement with the Italian law and the protocol was approved by an ethical committee of University of Perugia and by a National Committee of Italian Ministry of Health permits n° 1126/2016-PR and n. 583/2017-PR. The health and body conditions of the animals were monitored daily by the Veterinarian in the animal facility. 

### 2.11. Purification of Macrophages from the Spleen

The spleens were taken from AhR^+/+^ and AhR^−/−^ mice under sterile conditions. After homogenization, we performed the lysis of the red blood cells by hypotonic lysis. The cell pellet was resuspended in RPMI with 1% glutamine, 1% PS, and 20% of FBS at the concentration of 10^7^/mL. Macrophages were recovered by adherence to plastic after incubation for 2 h at 37 °C and 5% of CO_2_. The recovered macrophage-enriched cells were classically activated with LPS (5 ng/mL, Sigma-Aldrich L2880) + IFN-γ (20ng/mL, eBioscience, San Diego, CA, USA) for 16 h alone or in combination with P (20 µM) or Mt-P (20 µM) for the analysis of expression of several genes. 

### 2.12. Mice Model of Intestinal Inflammation

To investigate the effects of the two pelargonidins in a mouse model of intestinal inflammation, 8–10 weeks old male mice on a Balb/c and C57BL/6J (AhR wild type and AhR^−/−^ congenic littermates) genetic background were used. Mice were injected intrarectally with trinitrobenzenesulfonic acid (TNBS) (Sigma Chemical Co., St. Louis, MO, USA), as described elsewhere [24]. In brief, in TNBS-colitis model mice were fasted for 12 h overnight (day -1). The day after (day 0), under profound sedation induced by administration of mixture of tiletamine hypochoride and zolazepam hypocloride/xylazine at a dose of 50/5 mg/Kg, a 3.5 F = 11.55 mm catheter was inserted into the colon up to 4 cm from the anus and 1 mg of TNBS in 50% ethanol administered via the catheter into the colon lumen using a 1 mL syringe (injection volume of 100 μL). Control mice received 50% ethanol alone. At the end of the experiment, 4 days after the administration of TNBS, surviving mice were sacrificed, blood samples collected by cardiac puncture, and the colon excised, weighed, and evaluated for macroscopic damage. According to the experimental design, mice were administered orally pelargonidin (P) or Mt-P from 1 to 10 mg/kg/day, or dexamethasone, 5 mg/kg/day, by intraperitoneal injection (i.p.). The severity of colitis was scored daily in each mouse by assessing body weight, the fecal occult blood, and stool consistency. Each parameter was scored from 0 to 4 and the sum represents the Colitis Disease Activity Index (CDAI). The scoring system was as follows: Percent of body weight loss: None = 0; 1–5% = 1; 5–10% = 2; 10–20% = 3; >20% = 4. Stool consistency: Normal = 0; soft but still formed = 1; very soft = 2; diarrhea = 3; liquid stools that stick to the anus or anal occlusion = 4. Fecal blood: None = 0; visible in the stool = 2; severe bleeding with fresh blood around the anus and very present in the stool = 4.

### 2.13. Measurement of Intestinal Permeability

Two days before the sacrifice, a permeability test was carried out using a fluorescein isothiocyanate conjugated dextran (FITC-dextran) (Sigma-Aldrich, St. Louis, MO, USA, catalog number: FD4). FITC-dextran was dissolved in PBS at a concentration of 100 mg/mL and administered to each mouse at the dose of 44 mg/100 g body weight by oral gavage. After 4 h, mice were anesthetized by isoflurane inhalation and blood collected from the facial vein [25].

### 2.14. Isolation and Flow Cytometry Analysis of Colon Lamina Propria Cells

The cells were isolated from the colon lamina propria using the Lamina Propria Dissociation Kit (Miltenyi Biotec, Bergisch Gladbach, Germany; 130-097-401), according to the instructions.

Flow cytometry analyses were carried out using a two-laser standard configuration BD FACSVia™ flow cytometry system. Data were analyzed using FlowJo software (TreeStar). The gates were set using the fluorescence minus one (FMO) control strategy. FMO controls are samples that include all conjugated Abs present in the test samples except one. The channel in which the conjugated Ab is missing is the one for which the FMO provides a gating control. The following mAbs were used: CD4 PerCp-Cy5.5 (RM4-5, eBioscience, Affymetrix Santa Clara, CA, USA); CD11b Pe-Cy7 (M1/70, eBioscience, Affymetrix, Santa Clara, CA, USA); Gr1 PE (RB6-8C5, BioLegend, San Diego, CA, USA); IL-10 FITC (JES5-16E3, eBioscience, Affymetrix Santa Clara, CA, USA); and FoxP3 APC (FJK-16s, eBioscience, Affymetrix, Santa Clara, CA, USA).

### 2.15. NASH Model

In another experimental setting, Ahr^−/−^ male mice on a C57BL6/Sj background and their congenic littermates (AhR^+/+^) were fed a high fat diet (HFD) containing 59 KJ% fat plus 1% cholesterol, w/o sugar (ssniff^®^ EF R/M acc. D12330 mod. 22.7 ME/Kg) and fructose (HFD-F) in drinking water (42 g/L) or normal chow diet (5 and 7 mice, respectively) [26]. After 7 days, WT and Ahr^−/−^ HFD-F mice were randomized to receive HFD-F alone (6 and 8 mice, respectively) or in combination with Mt-P (5 mg/Kg/die) (6 and 9 mice, respectively) by gavage for 7 weeks. Mice were housed under controlled temperature (22 °C) and photoperiods (12:12-h light/dark cycle), and allowed unrestricted access to standard mouse chow and tap water. The experimental protocols were approved by the Animal Care and Use Committee of the University of Perugia and by the Italian Minister of Health and Istituto Superiore di Sanità (Italy), and were in agreement with the European guidelines for use of experimental animals (permission n. 1126/2016-PR and n. 583/2017-PR, respectively). The health and body conditions of the animals were monitored daily by the Veterinarian in the animal facility. On the day of sacrifice, fed mice were deeply anesthetized with a mixture of tiletamine hypochoride and zolazepam hypocloride/xylazine at a dose of 50/5 mg/Kg and sacrificed before 12 a.m. At the end of the study, the abdominal circumference (AC) (immediately anterior to the forefoot), body weight, and body length (nose-to-anus or nose–anus length) were measured in anesthetized mice at time of sacrifice. The body weight and body length were used to calculate the body mass index (BMI) (=body weight (g)/length^2^ (cm^2^)).

### 2.16. Biochemical Analyses, oral glucose tolerance test (OGTT), and Bile Acid Assay

Glucose, cholesterol, triglycerides, high density lipoproteins (HDLs), and low density lipoproteins (LDLs) plasmatic levels were quantified using an automated clinical chemistry analyzer (Cobas, Roche Basel, Switzerland). After 7 weeks of HFD-F, mice were fasted overnight and orally administered glucose (1.5 g/kg body weight) for OGTT. The blood glucose concentrations were measured at 0, 15, 30, 60, 90, and 120 min after feeding or injection using a portable glucose meter (Accu-Check Go, Roche, Basel, Switzerland) [27]. Gallbladder bile acids were quantified by a liquid chromatography-tandem mass spectrometry (MS/MS) analysis, as described elsewhere [28]. Briefly, the stock solutions of the individual tauro-conjugated and un-conjugated bile acids were prepared separately in methanol at a concentration of 1 mg/mL. All stock solutions were stored at −20 °C. Calibration standards were prepared by combining appropriate volumes of each bile acid stock solution and methanol. The calibration range was from 10 nM to 100 μM of each bile acid in the final solution. 10 mg of lyophilized gallbladder samples were manually pestled using a mortar and dissolved in 1 mL CH3OH. After centrifugation at 16,000× *g* for 10 min, 500 μL of supernatants were lyophilized and reconstituted in 100 μL of 40% water: 60% MeOH with 0.1% formic acid and ammonium acetate 5 mM. A bile acid extraction yield of 95% has been measured using bile acids standards addition in gallbladder samples before and after the deproteinization procedure [26].

### 2.17. Histology

For histological examination, samples of distal colon (2–3 cm from the anus), portions of the right and left liver lobes, and epididymal adipose tissue were fixed in 10% formalin, embedded in paraffin, sectioned, and stained with Sirius red and/or Hematoxylin/Eosin (H&E) for morphometric analysis. NASH severity (steatosis, hepatocytes ballooning, lobular inflammation, and portal inflammation) was scored in H&E-stained cross sections using an adapted grading system of human NASH, as described previously [29]. Hepatic fibrosis score was evaluated in Sirius red stained sections, as described previously [30]. 

### 2.18. Reverse Transcription of mRNA and Real-Time PCR

RNA was extracted from the liver, colon, spleen, epididymal adipose tissue (eWAT), and brown adipose tissue (BAT) using TRIzol reagent (Invitrogen) and Direct-zol™ RNA MiniPrep w/Zymo-Spin™ IIC Columns (Zymo Research, Irvine, CA, USA). After purification from genomic DNA using DNase I (Thermo Fisher Scientific, Waltham, MA, USA), 1 μg of RNA from each sample was reverse transcribed using random hexamer primers with SuperScript II (Thermo Fisher Scientific, Waltham, MA, USA) in a 20-μL reaction volume; 10 ng of cDNA was amplified in a 20-μL solution containing 200 nM of each primer and 10 μL of SYBR Select Master Mix (Thermo Fisher Scientific, Waltham, MA, USA). All reactions were performed in triplicate using the following thermal cycling conditions: 3 min at 95 °C, followed by 40 cycles of 95 °C for 15 s, 56 °C for 20 s, and 72 °C for 30 s, using a StepOnePlus system (Applied Biosystems, Foster City, CA, USA). The relative mRNA expression was calculated according to the ΔCt method. Primers were designed using the software PRIMER3 (http://frodo.wi.mit.edu/primer3/), using published data obtained from the NCBI database. Alternatively, for some genes, TaqMan probes (Thermo Fisher Scientific, Waltham, MA, USA) were used, with TaqMan GEX Master Mix (Thermo Fisher Scientific, Waltham, MA, USA). The primer used were as following (forward and reverse): *mGapdh* (for CTGAGTATGTCGTGGAGTCTAC; rev GTTGGTGGTGCAGGATGCATTG), *mIfn-γ* (for GCTTTGCAGCTCTTCCTCAT; rev ATCCTTTTGCCAGT), *mTnf-α* (for CCACCACGCTCTTCTGTCTA; rev AGGGTCTGGGCCATAGAACT), *mIl-6* (for CTTCACAAGTCGGAGGCTTA; rev TTCTGCAAGTGCATCATCGT), *mIl-1β* (for GCTGAAAGCTCTCCACCTCA; rev AGGCCACAGGTATTTTGTCG), *mTgf-β* (for TTGCTTCAGCTCCACAGAGA; rev TGGTTGTAGAGGGCAAGGAC), *mIl-10* (for CCCAGAAATCAAGGAGCATT; rev CTCTTCACCTGCTCCACTGC), *mF4/80* (for TGCAAAAGGATCCTCTTCAAGTG; rev ACTGGGGCACTTTTGTTCTCA), *mFoxP3* (for TCTTCGAGGAGCCAGAAGAG; rev AGCTCCCAGCTTCTCCTTTT), *mCd4* (for CACCTGGAAGTTCTCTGACCA; rev AAACGATCAAACTGCGAAGG), *mCd8* (for GCTCAGTCATCAGCAACTCG; rev GTGCACAGGTGAGGGAGTTC), *mCd11b* (for GTCAGAGTCTGCCTCCGTGT; rev CAGGGTCTAAAGCCAGGTCA), *mCd49b* (for CCATTCGCACCAAGTACTCC; rev ATAGCCATCCAGGGACCTTC), *mIl-10ra* (for GGTCGGAGGAGCAGTGTTTA; CCAGGATTCCACAGAATAGCA), *mIl-10rb* (for CATGGGCTTACAGAGTGCAA; rev GTAAGTTGTCCACGGCTCCA), *mUcp1* (for CTCACTCAGGATTGTGCCTCTAC; rev TCTGACCTTCACGACCTCTGTA), *mPgc-1α* (for CTTAGCACTCAGAACCATGCAG; rev AATGCTCTTCGCTTTATTGCTC), *mSrebp1c* (for GATCAAAGAGGAGCCAGTGC; rev TAGATGGTGGCTGCTGAGTG), *mFas* (for TCAAGATGAAGGTGGCAGAGGTGCT; rev TTGAGCAGTGCCGGGATTCGG), *mCd36* (for CGGAGACATGCTTATTGAGAA; rev ACTCTGTATGTGTAAGGACCT; *mPparα* (for CAGAGGTCCGATTCTTCCAC; GATCAGCATCCCGTGTTTGT), *mApoB100* (for GTCCTGGTCAACTTGCAAGC; rev GCTCATACCTTGTGTCCCCT); *mApoC* (for GCGTGCAGGAGTCCGATATA; rev GGTTAGAATCCCAGAAGCCG); *mCpt1α* (for TGCCAAATCTCTGCTGCATG; rev GGCCATGACATACTCCCACA), *mFgf21* (for CCTGGGTGTCAAAGCCTCTA; rev CTCCAGCAGCAGTTCTCTGA), *mAhR* (for ATCGCCACTCAGAGACCACT; rev ACAACACAGCCTCTCCGGTA), *mCyp1a1* (for ACTCTTCCCTGGATGCCTTC; rev GTCTGTGATGTCCCGGATGT), *mCyp1b1* (for TGTGGCTGCTCATCCTCTTT; rev GGTTGGGCTGGTCACTCAT); *hGAPDH* (for CAGCCTCAAGATCATCAGCA; rev GGTCATGAGTCCTTCCACGA), *hIL-6* (for CCAGGAGAAGATTCCAAAGATG; GGAAGGTTCAGGTTGTTTTCTG), *hBCL2* (for GAAACTTGACAGAGGATCATGC; TCTTTATTTCATGAGGCACGTT), *hIL-10R1* (for CCGAGAGTATGAGATTGCCATT; rev TCCAGAGGTTAGGAGGCTGA). The expression of marker genes for M1 and M2 macrophage populations were: *Cd38* (Mm01220906_m1 ThermoFisher Scientific), and *c-myc* (Mm00487804_m1 ThermoFisher Scientific).

### 2.19. Statistical Analysis

ANOVA One-way analysis of variance followed by a Tukey test or, alternatively, a two-tailed unpaired Student *t*-test were used for statistical comparisons (* *p* < 0.05) using Prism 6.0 software (GraphPad).

## 3. Results

### 3.1. Activity and Specificity of the Natural and Synthetic Pelargonidin Toward AhR 

**Figure 1:**
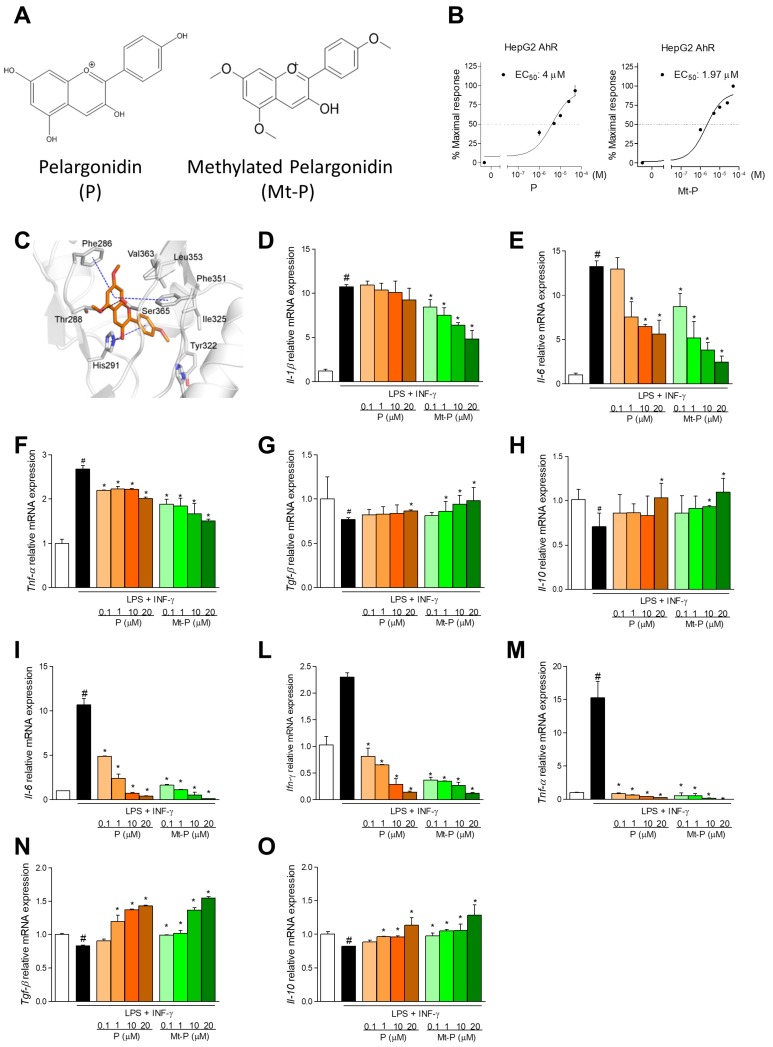
P and Mt-P counteracts in vitro macrophages and T-cells immune activation. The experiment was carried out on HepG2, Raw264.7 or EL4 cell lines. (**A**) Molecular structure of P and Mt-P. (**B**) Concentration-response curves of P and Mt-P on the aryl hydrocarbon receptor (AhR). HepG2 cells were transiently transfected as described in the Section 2. 24 h post-transfection cells were stimulated with increasing concentrations of P or Mt-P (1–50 µM). TCDD was used as a positive control. (**C**) Binding model of the Mt-P (orange sticks) into the PAS B domain of the AhR (white cartoon and sticks). Highlighted with dashed black line is the h-bond interaction with His291 and Ser 365, while with dashed blue line is the aromatic interaction. (**D**–**H**) In vitro effects of P and Mt-P on murine macrophages activation. Relative mRNA expression of pro- and anti-inflammatory genes was assessed by Real-Time PCR in Raw246.7 cells: (**D**) *Il-1β*, (**E**) *Il-6*, (**F**) *Tnf-α*, (**G**) *Tgf-β*, and (**H**) *Il-10* relative mRNA expression. (**I**–**O**) In vitro effects of P and Mt-P on murine T-cells activation. Relative mRNA expression of pro- and anti-inflammatory genes was assessed by Real-Time PCR in EL4 cells: (**I**) *Il-6*, (**L**) *Ifn-γ*, (**M**) *Tnf-α*, (**N**) *Tgf-β*, and (**O**) *Il-10*. Values are normalized relative to *Gapdh* mRNA and are expressed as mean ± SEM (*n* = 3); ^#^ *p* < 0.05 vs. NT cells, * *p* < 0.05 vs. LPS/IFN-γ cells.

The natural pelargonidin (Figure 1A, Pelargonidin P) was obtained by the extraction from red radish peels (European variety). The extract was purified by silica gel in reverse phase and the anthocyanins fraction was collected and dried under N_2_ flux. The pelargonidins purified extract was hydrolyzed in acidic conditions and purified to obtain the aglycon form (see Section 2.2 in Materials and Methods). The methylated pelargonidin (Figure 1A, Mt-P) does not exist in nature, and for this reason it was obtained by de novo synthesis (Scheme 1, Materials and Methods). We first investigated whether the natural (P) and synthetic pelargonidin (Mt-P) (chemical structures shown in Figure 1A) exerted agonistic effects on a family of ligand-activated nuclear receptors, including AhR (Figure S1A,B), PXR, FXR, LXRα, and β, and PPARs and on the G protein-coupled receptor, GPBAR1 (Figure S1C–F). 

For this purpose, HepG2 cells, transfected with an AhR reporter gene cloned upstream to the luciferase, were incubated with 5 nM TCDD, an AhR agonist as a control, or vehicle (0.1% v/v DMSO) in the presence of the two pelargonidins (0.1–100 µM) for 18 h. As shown in Figure 1B and Figure S1, both anthocyanins were effective in transactivating the AhR, while they exerted no effect on the other receptors (Figure S1C–F, * *p* < 0.05 versus control cells). Both pelargonidins produced a concentration-dependent increase of luciferase activity, with a relative potency, expressed as EC_50_, of 4 and 1.97 µM for the natural pelargonidin and Mt-P, respectively (Figure 1B). The interaction of Mt-P with the PAS-B domain of AhR was further investigated by in silico analysis. AhR is a cytosolic signal sensor protein with a multi-modular structure, composed by an amino N-ter domain, namely bHLH domain, which is responsible for DNA binding, two PER-ARNT-SIM domains (PAS A and PAS B), and a carboxy C-ter transactivation domain (TAD). It has been experimentally demonstrated that AhR binds chemically different exogenous ligands at the PAS-B domain [31,32,33,34]. In order to get preliminary clues about the mode of interaction between the synthetic pelargonidin, we performed a set of docking studies. Unfortunately, the structure of the PAS B domain of AhR is not yet available, therefore we generated the homology 3D model structure of such a domain, which was used to carry on the docking studies. Figure 1C shows the binding mode of the Mt-P into the PAS B domain of AhR. Key interactions are represented by: (i) Two T-π hydrophobic interaction with His291 and Phe351 (blue dashed lines); (ii) a parallel displaced interaction with Phe286 (blue dashed line); (iii) hydrophobic interaction with Thr288, Tyr322, Ile325, Leu353, Val363; and (iv) two h-bonds with His291 and Ser365 (black dashed line). Interestingly, the ability of the Met-P to interact with His291 and Ser365 agrees with experimental data showing that the agonistic effects of AhR binders is mediated by the interaction with these two keys residues [31,32,34].

### 3.2. In Vitro Characterization of Mt-P on Macrophages and T Cells

We then investigated whether the Mt-P exerts AhR-dependent activities in vitro. For this purpose, RAW264.7 cells were primed with LPS and IFN-γ, and co-incubated with or without increasing concentrations of the two pelargonidins. As shown in Figure 1D–F, both pelargonidins were somewhat effective in attenuating the production of pro-inflammatory cytokines (Il-1β, Il-6, Tnf-α) in this experimental setting. However, while the natural pelargonidin exerted no effect on Il-1β and caused only a slight reduction of Tnf-α gene transcription, the Mt-P blunted the expression of these inflammatory mediators by 50 to 80% (Figure 1D–F, ^#^ *p* < 0.05 vs. NT cells, * *p* < 0.05 vs. LPS/IFN-γ cells). Additionally, exposure to the Mt-P increased the expression of anti-inflammatory genes Tgf-β and Il-10, while the natural agent exerted no effect or was only minimally effective (Figure 1G,H, ^#^ *p* < 0.05 vs. NT cells, * *p* < 0.05 vs. LPS/IFN-γ cells). 

In contrast to the relatively minor effects exerted by the natural pelargonidin on macrophages, both P and Mt-P were extremely potent in attenuating cytokines generation by T cells (Figure 1I–O). Thus, while priming EL4 T cells with LPS/INF-γ increased the expression of *Il-6, Ifn-γ* and *Tnf-α* (Figure 1I–M, ^#^ *p* < 0.05 vs. NT cells, * *p* < 0.05 vs. LPS/IFN-γ cells) and reduced the expression of *Tgf-β* and *Il-10* (Figure 1N–O, ^#^ *p* < 0.05 vs. NT cells, * *p* < 0.05 vs. LPS/IFN-γ cells), these effects were completely reversed by both agents in a concentration-dependent manner. 

**Figure 2:**
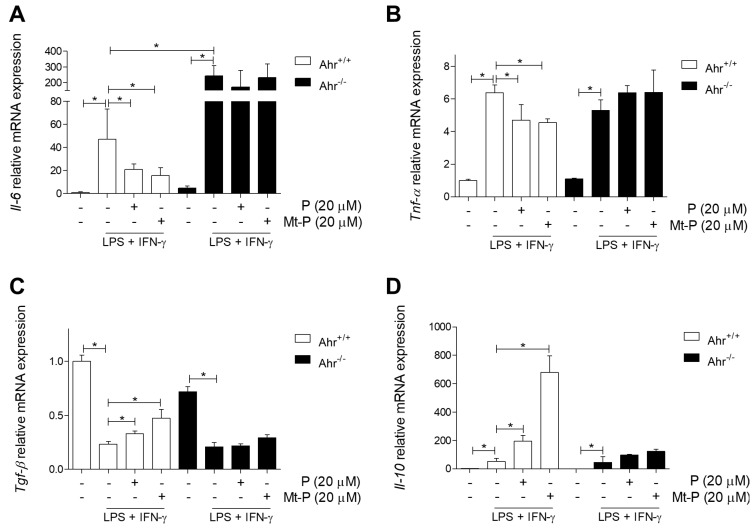
P and Mt-P counteracts in vitro macrophages immune activation through the AhR. The experiment was carried out on macrophages purified by the spleen of AhR^+/+^ and AhR^−/−^ mice. Macrophages cells were primed 16 h with LPS (5 ng/mL)/IFN-γ (20 ng/mL) alone or in combination with P (20 µM) and Mt-P (20 µM). At the end of stimulation, the relative mRNA expression of pro-inflammatory cytokines (**A**) *Il-6* and (**B**) *Tnf-α*, and anti-inflammatory cytokines (**C**) *Tgf-β* and (**D**) *Il-10*, was assessed by Real-Time PCR. Values are normalized relative to *Gapdh* mRNA and are expressed as mean ± SEM (*n* = 4); * *p* < 0.05.

To further investigate whether these immune-modulatory effects were mediated by binding/activation of the AhR, spleen-derived macrophages isolated from AhR^+/+^ and AhR^−/−^ mice were incubated with LPS/IFN-γ alone or in combination with the two pelargonidins (Figure 2). Once again, both agents reversed the generation of pro-inflammatory mediators caused by exposure to LPS/IFN-γ (Figure 2A,B, ^#^ *p* < 0.05 vs. NT cells, * *p* < 0.05 vs. LPS/IFN-γ cells), while induced an up-regulation of the anti-inflammatory cytokines *Il-10* and *Tgf-β*, but this effect was completely abrogated by AhR gene ablation (Figure 2A–D, ^#^ *p* < 0.05 vs. NT cells, * *p* < 0.05 vs. LPS/IFN-γ cells). 

### 3.3. In Vitro Characterization of Mt-P on Intestinal Epithelial Cells

**Figure 3:**
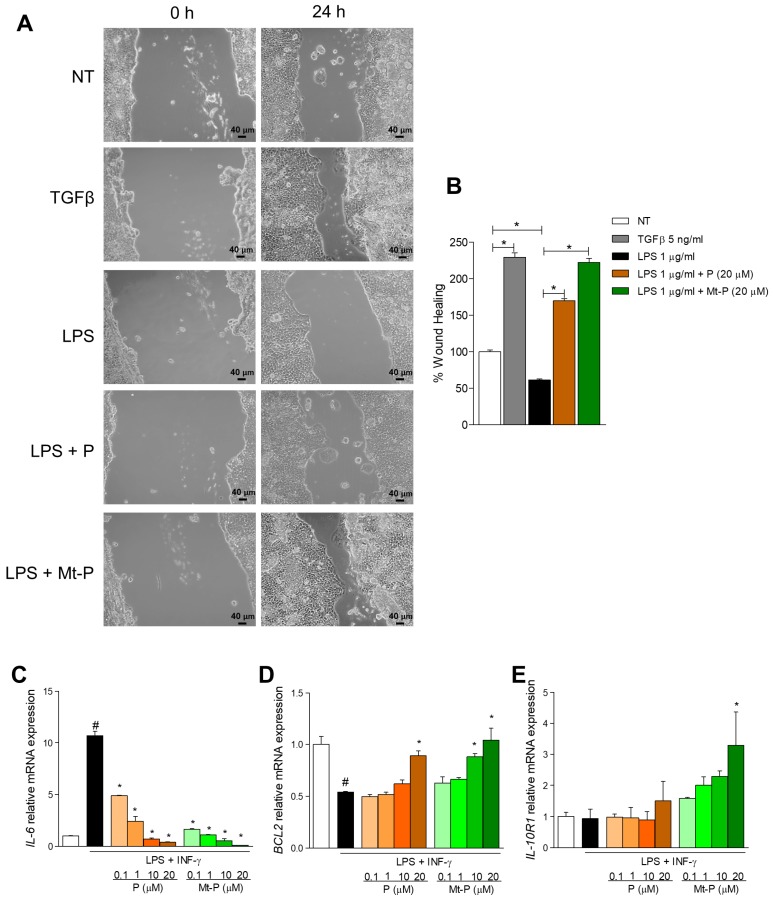
P and Mt-P accelerates the wound healing in Caco-2 cells, reducing inflammation and apoptosis. Confluent Caco-2 cell monolayers were wounded with a pipette tip and incubated with medium alone (NT), TGFβ (5 ng/mL) used as positive control, LPS (1 µg/mL) alone or in combination with P and Mt-P (20 µM) for 24 h. (**A**) Representative images of scratched areas at 0 and 24 h (Magnification 10×). (**B**) Percentage of wound healing at 24 h after scratch (five wells of three fields each) (NT wells were arbitrarily assigned as 100%). Wound healing was photographed at 0 and 24 h after scratch. Results are expressed as mean ± S.E.M. and analyzed using ANOVA followed by Bonferroni’s test. * *p* < 0.05 compared to control group. (**C**–**E**) In vitro effects of P and Mt-P on human intestinal epithelial cell activation. Serum-starved Caco-2 cells were primed 16 h with LPS (5 ng/mL)/IFN-γ (20 ng/mL) alone or in combination with increasing concentration of both P and Mt-P (0.1–20 µM). At the end of stimulation, the relative mRNA expression of pro-inflammatory gene *IL-6*, anti-apoptotic gene *BCL-2*, and *IL-10R1*, was assessed by Real-Time PCR. Values are normalized relative to *GAPDH* mRNA and are expressed relative to those of not treated cells (NT), which are arbitrarily set to 1 (*n* = 3, ^#^ *p* < 0.05 vs. NT, * *p* < 0.05 vs. LPS).

Since intestinal epithelial cells express AhR and the receptor is involved in maintenance of intestinal barrier function, we then investigated whether P and Mt-P promote CaCo-2 cells migration in an in vitro model of wound healing, an indirect measure of the ability of intestinal epithelial cells to repair mucosal injuries and maintain intestinal barrier integrity. As shown in Figure 3A,B, treating CaCo2 cells with LPS impaired the wound closure in this model. Conversely, Mt-P reversed the effect of LPS, greatly accelerating the wound closure to the same extent of TGF-β, a potent inducer of CaCo2 cell migration, that was used as positive control (Figure 3A,B, ^#^ *p* < 0.05 vs. NT cells, * *p* < 0.05 vs. TGF-β cells).

To gain some mechanistic insights into this model, we assessed the effect of P and Mt-P on the expression of *IL-6, BCL2*, and *IL-10R1* genes in CaCo2 cells exposed to LPS/IFN-γ, and found that bot agents completely abrogated *IL-6* mRNA transcription in a concentration-dependent manner (Figure 3C, ^#^ *p* < 0.05 vs. NT cells, * *p* < 0.05 vs. LPS/IFN-γ cells). Moreover, Mt-P increased the expression of the anti-apoptotic gene *BCL2* (Figure 3D, ^#^ *p* < 0.05 vs. NT cells, * *p* < 0.05 vs. LPS/IFN-γ cells), as well expression of *IL-10R1* (Figure 3E, ^#^ *p* < 0.05 vs. NT cells, * *p* < 0.05 vs. LPS/IFN-γ cells) mRNA, a gene encoding the interleukin 10 receptor 1, which is known to induce the proliferation of intestinal epithelial cells [35].

### 3.4. Mt-P Rescues from Intestinal Inflammation in an AhR-Dependent Manner

We then investigated whether pelargonidins attenuate intestinal inflammation and immune dysfunction in a mouse model of colitis, induced by treating BALB/c mice with TNBS. The results shown in Figure S2 demonstrate that the development of clinical signs and symptoms of intestinal inflammation and their severity were greatly attenuated by treatment with both P and Mt-P, though only the effect of Mt-P was dose-dependent over the range of concentrations tested. Indeed, while doses of 5–10 mg/kg P were required to prevent development of all clinical signs and symptoms of colitis (Figure S2C–F; ^#^ *p* < 0.05 vs. NT mice, * *p* < 0.05 vs. TNBS mice), a dose of 5 mg/kg of Mt-P successfully reversed inflammatory changes caused by TNBS (Figure S2, ^#^ *p* < 0.05 vs. NT mice, * *p* < 0.05 vs. TNBS mice). 

**Figure 4:**
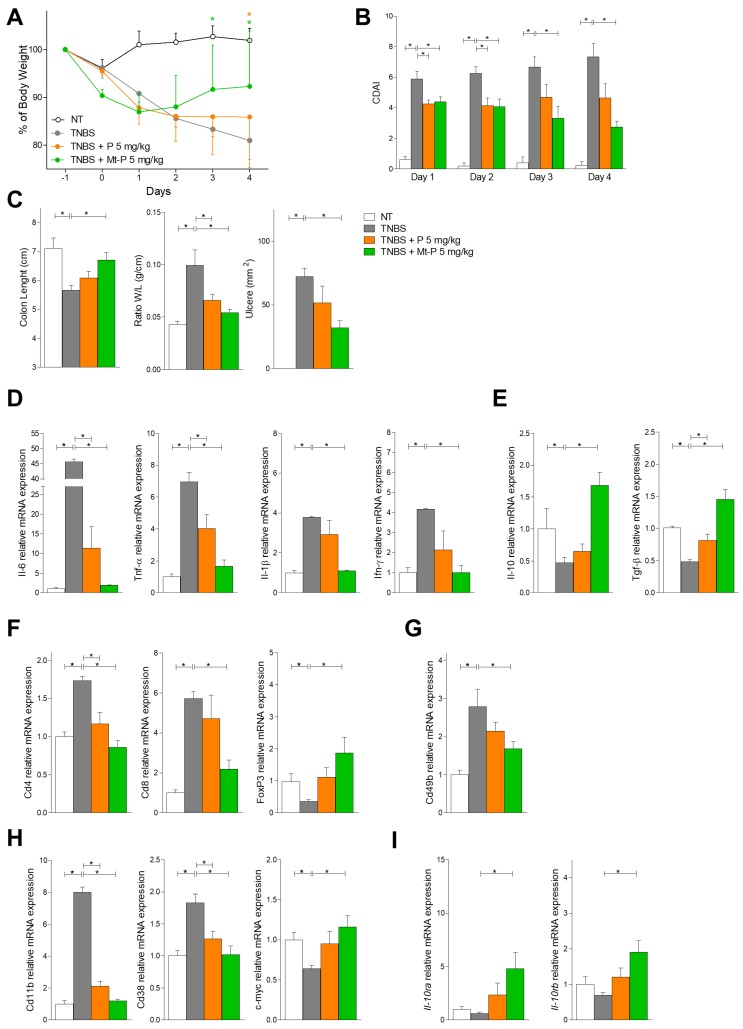
Mt-P is more effective than P in reducing the severity of colitis. Balb/C mice were treated with TNBS alone or in combination with P or Mt-P (5 mg/kg) as described before (see Section 2). (**A**) Changes in body weight and (**B**) Colitis Disease Activity Index (CDAI) of mice during the course of TNBS-induced colitis. (**C**) Analysis of the intestinal inflammatory score: Colon length (**left**), ratio of colon weight/colon length (**central**), and ulcers (**right**). Relative mRNA expression of pro-inflammatory cytokines (**D**) *Il-6, Tnf-α, Il-1β*, and *Ifn-γ*, (**E**) anti-inflammatory cytokines *Il-10* and *Tgf-β*, (**F**) T cell markers *Cd4, Cd8*, and *FoxP3*, (**G**) NK and NKT markers *Cd49b*, (**H**) macrophages markers *Cd11b, Cd38*, and *c-myc*, and (**I**) *Il-10ra* and *Il-10rb* genes in the colons of mice assessed by real-time PCR. Values are normalized relative to *Gapdh* mRNA and are expressed as mean ± SEM of five/eight mice per group (NT = 5; TNBS = 8; TNBS + P 5 mg/kg = 6; TNBS + Mt-P 5 mg/kg = 8) (* *p* < 0.05).

Because these data suggest that 5 mg/kg of Mt-P was fully effective in reversing signs of intestinal inflammation, we investigated the mechanisms involved in the beneficial effects exerted by this agent, in comparison to the natural pelargonidin, on intestinal immunity. Again, as shown in Figure 4, Mt-P at the dose of 5 mg/kg was significantly more effective than P in protecting from appearance of the clinical signs of colitis (body weight loss and CDAI), and was significantly more effective in attenuating macroscopic changes caused by TNBS (Figure 4A–C, * *p* < 0.05). Additionally, while treating mice with TNBS increased the colonic expression of *Il-6, Tnf-α, Il-1β* and *Ifn-γ*, (Figure 4D, * *p* < 0.05), this pattern was fully reversed by treatment with the Mt-P, that also increased the colonic expression of *Tgf-β* and *Il-10* mRNA (Figure 4E, * *p* < 0.05). 

Amelioration of macroscopic signs of colitis correlated with a robust attenuation of inflow of inflammatory cells in the colonic lamina propria, as shown in Figure 4F–H. Again, while treatment with Mt-P significantly reduced the polarization of colonic cell infiltrates toward a pro-inflammatory phenotype, as assessed by measuring the levels of expression of *Cd11b, Cd8, Cd4, CD49b*, and *Cd38*, this pattern was only partially reproduced by the natural pelargonidin. Additionally, as shown in Figure 4F–I, Mt-P, but not the natural pelargonidin, increased the expression of *c-myc*, a M2 marker, and *FoxP3*, a Treg marker (Figure 4F–I, * *p* < 0.05). The later effects were not observed in mice treated with the natural pelargonidin.

**Figure 5:**
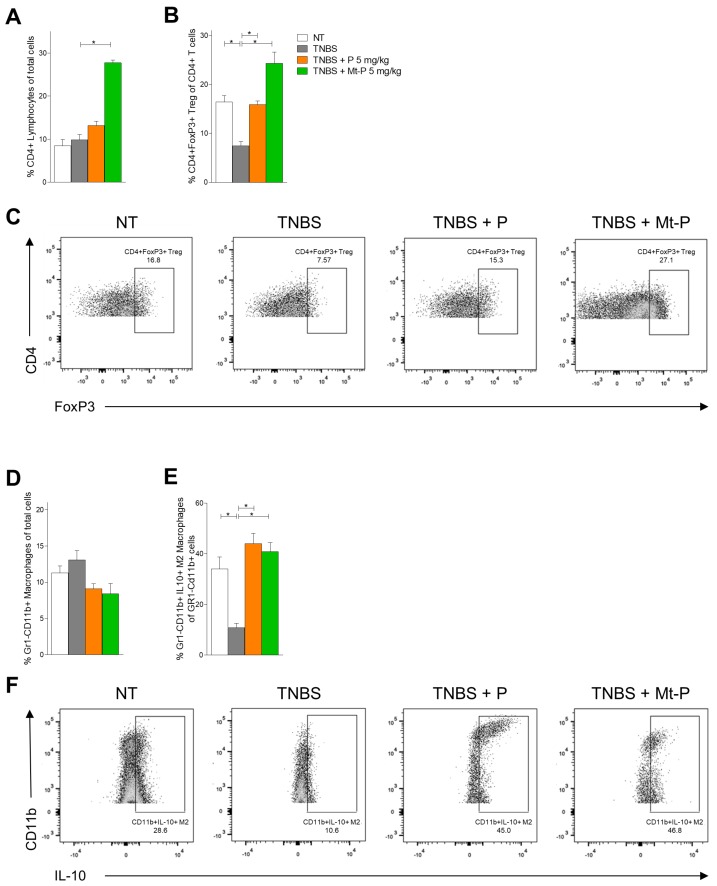
Effect of P and Mt-P on the polarization of the LP immune cells in the TNBS model of colitis. Frequency of immune cells in the colonic lamina propria (LP): (**A**) CD4+ cells and (**B**) CD4+FoxP3+ cells. (**C**) Flow cytometry analysis of FoxP3 expression in CD4+ cell recruited into the LP. Frequency of (**D**) CD11b+Gr1-macrophages and (**E**) CD11b+Gr1-IL-10+ M2 macrophages in the colonic LP. (**F**) Flow cytometry analysis of Il-10 expression in CD11b+Gr1-macrophages recruited into the LP. Results are the mean ± SEM of five/eight mice per group (NT = 5; TNBS = 8; TNBS + P 5 mg/kg = 6; TNBS + Mt-P 5 mg/kg = 8). (* *p* < 0.05).

These findings were confirmed by a flow-cytometry analysis of colonic lamina propria infiltrating cells (Figure 5A–C), demonstrating that protection exerted by Mt-P associated with, and increased recruitment of, CD4+ FoxP3+ (Treg cells) in the lamina propria. Furthermore, analysis of macrophage subsets demonstrated that the in vivo exposure to the two agents increased the polarization of colonic macrophages toward a Cd11b+/IL-10+ anti-inflammatory phenotype (Figure 5D–F). 

**Figure 6:**
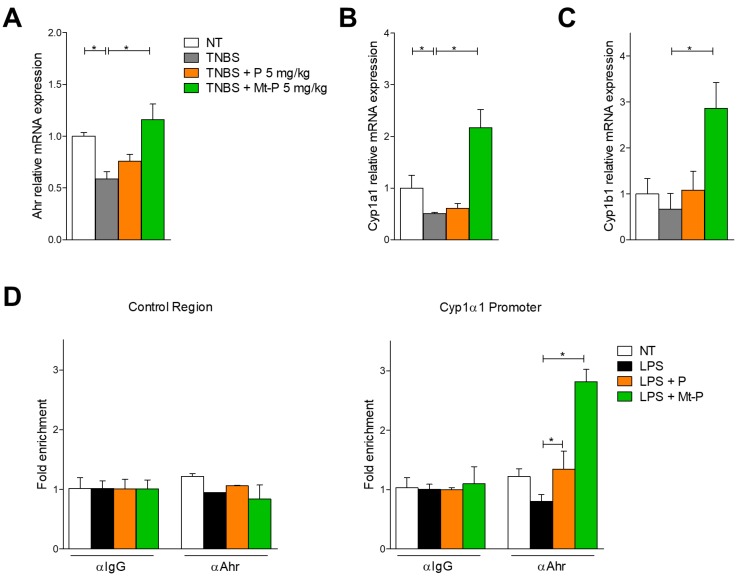
P and Mt-P activate the aryl hydrocarbon receptor (AhR). Relative mRNA expression of (**A**) *AhR*, (**B**) *CYP1a1*, and (**C**) *CYP1b1* genes in the colons of mice treated with TNBS alone or in combination with P or Mt-P, assessed by real-time PCR. Data are normalized to *Gapdh* mRNA. Results are the mean ± SEM of five/eight mice per group (NT = 5; TNBS = 8; TNBS + P 5 mg/kg = 6; TNBS + Mt-P 5 mg/kg = 8) (* *p* < 0.05). (**D**) ChIP assay of the AhR binding *Cyp1a1* promoter in Raw264.7 cells. Cell lysates were immunoprecipitated with anti-AhR or control IgG, and the presence of specific regions in the immune-precipitates was determined by RT-PCR. (*n* = 3) (* *p* < 0.05).

To investigate whether the mechanism of action of P and Mt-P in vivo requires an intact AhR signaling, we then assessed the expression of the receptor, along with AhR-regulated genes in the colons of mice treated with TBNS and found that, while TNBS administration decreased the colonic expression of AhR mRNA and its target genes *Cyp1a1* and *Cyp1b1*, this pattern was reversed by treating mice with Mt-P, while the natural compound was ineffective (Figure 6A–C, * *p* < 0.05).

To further elaborate on the mechanism involved in the regulation of AhR-regulated genes in vivo, we assessed whether exposure to P and Mt-P resulted in the occupation of *Cyp1a1* promoter by the AhR. Because the analysis of *Cyp1a1* promoter revealed the presence of binding sequences for the AhR/ARNT dimer (see Section 2), we carried out a ChIP assay on RAW264.7 cells to investigate whether exposure to P and Mt-P increased the binding of the AhR/ARNT dimer to the *Cyp1a1* promoter. As shown in Figure 6D, while exposure to LPS slightly reduced the occupation of *Cyp1a1* promoter by the AhR/ARNT dimer, this effect was reversed by both P and Mt-P, but the synthetic compound was significantly more effective and increased the binding of the AhR/ARNT dimer to the *Cyp1a1* promoter by almost three-fold (Figure 6E, * *p* < 0.05 vs. LPS treated cells).

**Figure 7:**
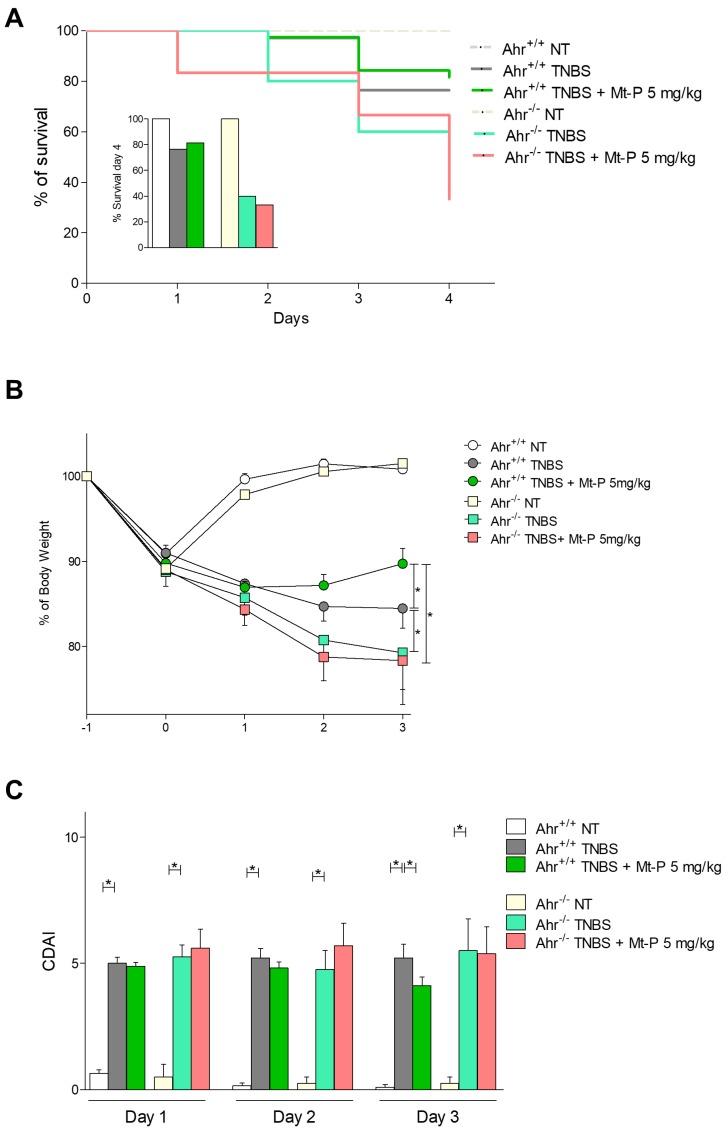
The beneficial effect of Mt-P is lost in AhR^−/−^ mice. AhR^+/+^ and AhR^−/−^ mice were treated with 1 mg of TNBS and then administered vehicle or Mt-P (5 mg/kg/d) by gavage from days 0 to 4. Treatment with Mt-P attenuated the development of disease only in AhR^+/+^ mice, on the contrary AhR gene ablation aggravated the disease. The disease was monitored by (**A**) percentage of survival, (**B**) changes in body weight, and (**C**) CDAI score. Results are the mean ± SEM of five/twelve mice per group (Ahr^+/+^ NT = 8; Ahr^+/+^ TNBS = 10; Ahr^+/+^ TNBS + Mt-P 5 mg/kg = 12; Ahr^−/−^ NT = 5; Ahr^−/−^ TNBS = 5; Ahr^−/−^ TNBS + Mt-P 5 mg/kg = 6) (* *p* < 0.05).

We next investigated whether the anti-inflammatory activities of Mt-P are maintained in AhR-knockout mice. For this purpose, wild-type and AhR^−/−^ mice on a C57BL/6 background were administered TNBS (0.5 or 1 mg/mice), with or without Mt-P (5 mg/kg). As shown in Figure 7, the severity of disease was exacerbated by AhR gene ablation. In comparison with their congenic littermates, AhR^−/−^ mice developed an aggressive disease associated with a high rate mortality on day 4 (70% versus 25%), as shown in Figure 7A. Furthermore, while treatment with Mt-P protected from colitis development in AhR^+/+^ mice, the protective effects were lost in AhR^−/−^ mice (Figure 7B,C). 

Since glucocorticoids are widely used in the treatment of IBDs, we compared the beneficial effects of Mt-P to dexamethasone in the treatment of colitis induced by TNBS. The data shown in Figure S3A,B, demonstrate that both Mt-P and dexamethasone effectively attenuated the development of wasting disease and disease severity (Figure S3B, * *p* < 0.05), and exerted similar beneficial effects on colon length, colon weight/length ratio, and number of ulcerations (Figure S3C–E, * *p* < 0.05).

### 3.5. Mt-P Attenuates Intestinal Inflammation and Insulin Resistance in Mice Fed a HFD-F

**Figure 8:**
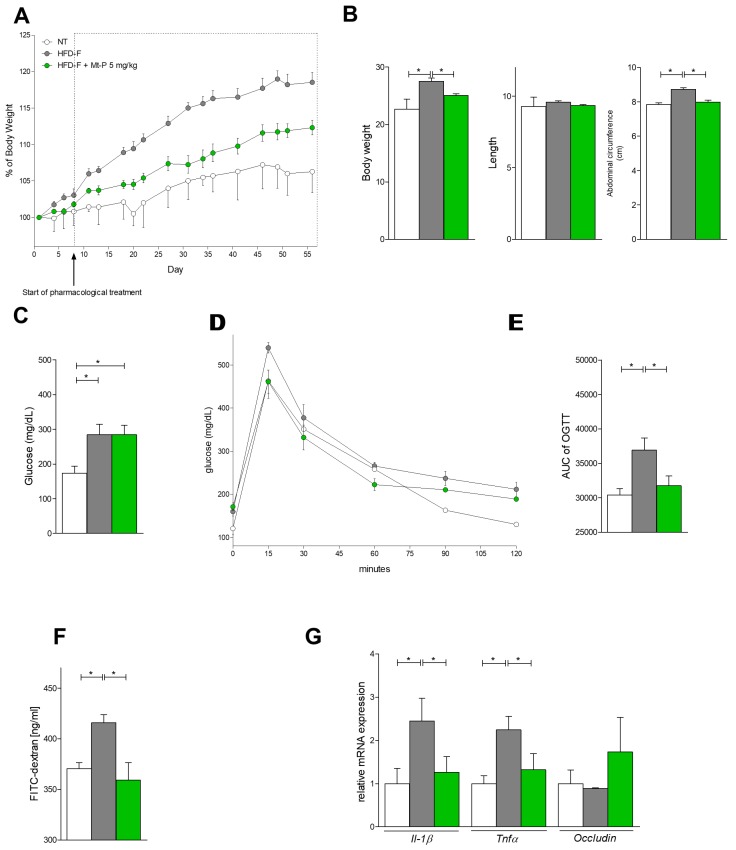
Activation of AhR reduced body weight gain and insulin resistance in mice fed a HFD-F. C57BL/6 male mice were fed a high fat diet (HFD) and fructose or normal chow diet as described before (see Section 2). (**A**) Changes in body weight (%) assessed weekly. Anthropometrical parameters: (**B**) Body mass index (BMI) measured at the end of the study; (**C**) glucose plasma levels; (**D**) glucose levels in response to OGTT; and (**E**) Air Under the Curve (AUCs) of glucose plasma levels expressed in arbitrary units. Results in intestine: (**F**) Intestinal permeability was measured after 2 weeks of diet with FITC-dextran; and (**G**) quantitative RT-PCR analysis of *Il-1β*, *Tnfα*, and *occludin1*. Values are normalized relative to *Gapdh* mRNA and are expressed as mean ± SEM of six/ten mice per group (NT = 6; HFD-F = 8; HFD-F + Mt-P 5 mg/kg = 10) (* *p* < 0.05).

To test whether therapeutic potentials of Mt-P extended beyond the intestine, we set up a model of intestinal inflammation in the frame of metabolic dysfunction caused by feeding mice HFD-F for 8 weeks. As shown in Figure 8, mice exposed to chronic high caloric intake gained significantly more body weight than mice feed a normal chow diet, and they had significantly higher BMI at the end of the study (Figure 8A,B, * *p* < 0.05). 

Treating mice with 5 mg/kg/day Mt-P for 8 weeks protected against body weight gain and resulted in a lower BMI in comparison to mice feed a HFD-F (Figure 8A,B, * *p* < 0.05). After 8 weeks of feeding a HFD-F, mice developed insulin resistance, as demonstrated by higher glucose plasma levels, and altered glucose levels in response to OGTT in comparison to naïve mice (Figure 8C–E). Treating mice with Mt-P effectively improved the glucose tolerance test and reduced the Air Under the Curve (AUC) of OGTT (Figure 8D,E, * *p* < 0.05). 

Analysis of intestine functionality in this model demonstrated that chronic exposure to a high caloric intake increased dramatically the intestinal permeability (Figure 8F), as measured by assessing plasma levels of the FITC dextran at the end of the study, along with expression of colonic markers of inflammation such as *Il-1β* and *Tnf-α* (Figure 8G). These changes were completely reversed by treating HFD-F mice with Mt-P (Figure 8F,G, * *p* < 0.05). While no effects were observed in HFD-F mice, Mt-P administration increased the expression of *occludin* mRNA, thus confirming a decreased intestinal permeability (Figure 8F).

**Figure 9:**
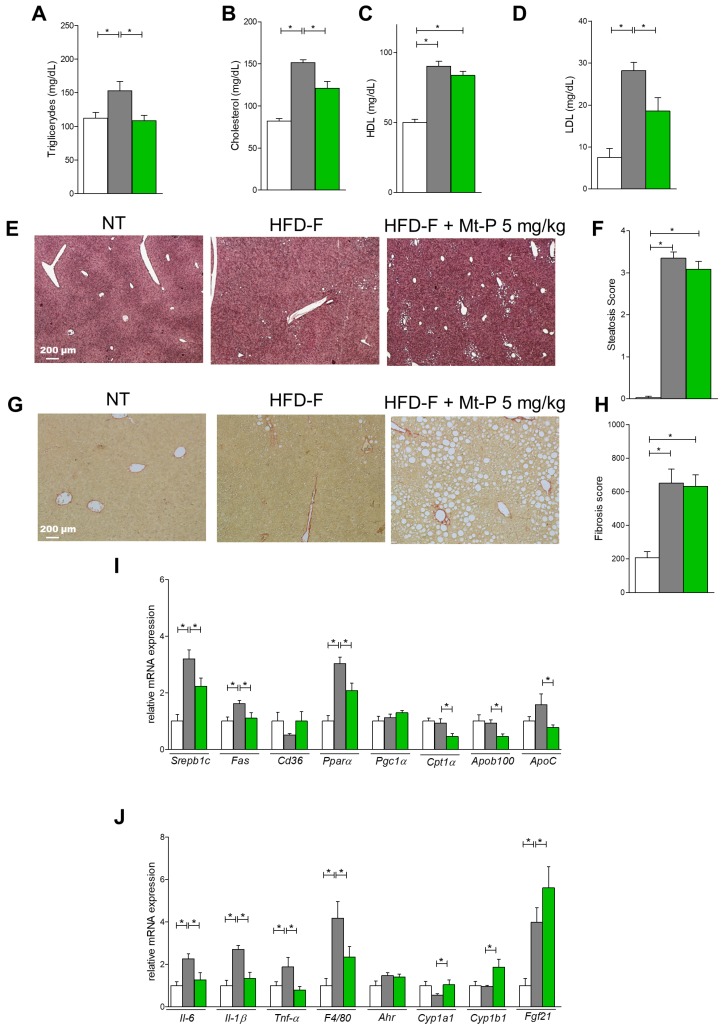
AhR activation exacerbated NASH-like features and fibrosis induced by HFD-F exposure. C57BL/6 male mice were fed a high fat diet (HFD) and fructose or normal chow diet as described before (see Section 2). Serum levels of (**A**) cholesterol, (**B**) triglycerides, (**C**) high density lipoproteins (HDLs), and (**D**) low density lipoproteins (LDLs). Biochemistry values were measured at the end of the study. (**E**) Hematoxylin and eosin (H&E) staining of liver tissues obtained at the end of the study (Magnification 4×). NASH severity was scored by assessing the (**F**) steatosis (NAS) score in at least five different fields per liver in a blind manner. (**G**) Sirius red staining of liver tissues obtained at the end of the study (Magnification 10×). (**H**) Liver fibrosis score (% collagen deposition) was measured, as described in Material and Methods, by ImageJ analysis. The images shown in panels (**E**–**H**) are representative of at least 10 others, each one obtained from an individual mouse, showing a similar pattern of regulation. Hepatic expression of (**I**) genes involved in the regulation of lipid synthesis (*Srebp1c, Fas*), fatty acids oxidation (*Pparα, Pgc1α, Cpt1α*), and very low-density lipoprotein (VLDL) formation (*Apo100B, ApoC*), and (**J**) pro-inflammatory genes (*Il-6, Tnfα, Il-1β, F4/80*) and Ahr-target genes (*Ahr, Cyp1a1, Cyp1b1, Fgf21*). Values are normalized relative to Gapdh mRNA and are expressed as mean ± SEM of six/ten mice per group (NT = 6; HFD-F = 8; HFD-F + Mt-P 5 mg/kg = 10) (* *p* < 0.05).

We then characterized the effects of Mt-P on plasma and liver biochemistry in mice feed HFD-F. As shown in Figure 9, at the time of sacrifice, mice feed a HFD-F alone had increased plasma levels of triglycerides and cholesterol (Total HDL and LDL) (Figure 9A–D, * *p* < 0.05). This pattern was reversed by Mt-P, with the exception of HDL cholesterol (Figure 9C, * *p* < 0.05).

Mice challenged with HFD-F for 8 weeks developed NASH-like features, as demonstrated by H&E staining of liver sections, with micro-vesicular steatosis and hepatocytes ballooning (Figure 9E), which resulted in a marked increase in the liver steatosis score (Figure 9F, * *p* < 0.05). Furthermore, exposure to a HFD-F increased liver collagen deposition, resulting in moderate fibrosis, as shown by scoring Sirius red stained liver sections (Figure 9G,H, * *p* <0.05). To some surprise, despite an improvement in body weight and insulin resistance, treating HFD-F mice with Mt-P worsened lipid accumulation, resulting in severe steatosis as shown by H&E staining, with no deterioration of liver fibrosis (Figure 9E–H, * *p* < 0.05). Treating mice with Mt-P failed to ameliorate liver steatosis and fibrosis (Figure 9E–H, * *p* < 0.05).

Since liver steatosis in mice feed HFD-F is the result of impaired triglycerides synthesis, fatty acid oxidation, or lipid uptake, we then investigated the expression of genes involved in the regulation of these pathways. Interestingly, as shown in Figure 9I, mice treated with Mt-P had a reduced expression of lipogenic genes such as *Srebp1c* and *Fas*, compared with HFD-F mice (Figure 9I, * *p* < 0.05), while the expression of fatty acid oxidation genes, including *Pparα* and its target gene *Cpt1α*, was significantly decreased in HFD-F mice administered the AhR ligand. Furthermore, AhR activation in the liver resulted in an impaired triglyceride secretion by hepatocytes, as indicated by reduced expression of genes involved in very low-density lipoprotein (VLDL) formation, such as *Apob100* and *ApoC* (Figure 9J, * *p* < 0.05). The analysis of inflammatory markers revealed that mice on HFD-F had an increased liver expression of pro-inflammatory genes such as *Il-6, Tnfα, Il-1β* (Figure 9J). This pattern was attenuated by treating mice with Mt-P (Figure 9J, * *p* < 0.05). Moreover, Mt-P increased the expression of *AhR* and its target genes, including *Cyp1a1, Cyp1b1*, and *Fgf21* (Figure 9J, * *p* < 0.05), confirming the in vivo efficacy of the compound.

**Figure 10:**
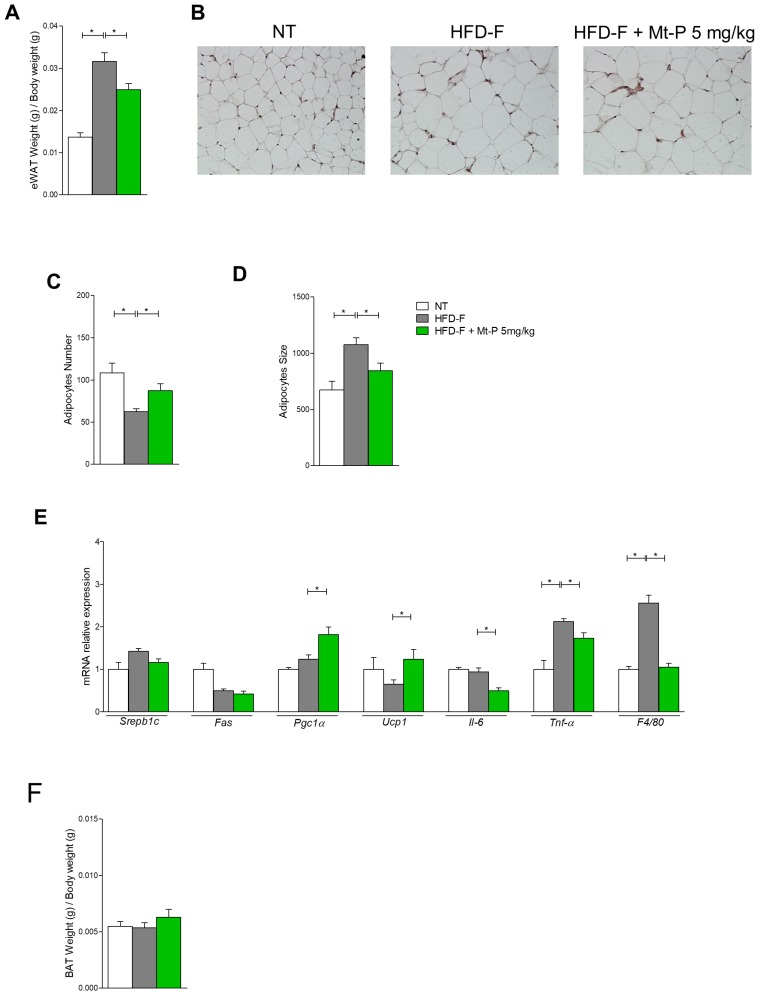
Effects of AhR activation in extrahepatic tissues. C57BL/6 male mice were fed a high fat diet (HFD) and fructose or normal chow diet as described before (see Section 2). (**A**–**C**)**:** Results in epididymal white adipose tissue (eWAT). (**A**) Ratio eWAT weight/body weight. (**B**) Hematoxylin and eosin (H&E) staining of white adipose tissue sections obtained at the end of the study. The images shown are representative of at least 10 others, each one obtained from an individual mouse, showing a similar pattern of regulation (magnification 4×). (**C**) Adipocytes number and (**D**) size obtained from analysis of H&E staining performed with ImageJ. (**E**) Changes in mRNA expression of eWAT genes. (**F**) Ratio brown adipose tissue (BAT) weight/body weight. Values are normalized relative to Gapdh mRNA and are expressed as mean ± SEM of six/ten mice per group (NT = 6; HFD-F = 8; HFD-F + Mt-P 5 mg/kg = 10) (* *p* < 0.05).

We then investigated the metabolic effects of AhR activation in metabolic tissues, including the epididymal white adipose tissue (eWAT). As shown in Figure 10, exposure to the HFD-F increased the eWAT weight (Figure 10A) and induced several morphological changes, including a reduction of the number of adipocytes, while increased their size, as revealed by the analysis of H&E staining sections (Figure 10B–D, * *p* < 0.05). 

Co-treating mice with the Mt-P reversed this pattern—i.e., reduced eWAT weight (as illustrated in Figure 10A) and restored adipocyte morphology (Figure 10B–D, * *p* < 0.05). Because obesity is associated with an increase in macrophage infiltration in WAT, we then measured the expression of pro-inflammatory markers in eWAT. As shown in Figure 10E, while feeding a HFD-F increased expression of *F4/80* and *Tnf-α*, this pattern was reversed by Mt-P (Figure 10E, * *p* < 0.05). Finally, treating HFD-F mice with Mt-P resulted in an increased expression of *Pgc1α* and *Ucp1*, two genes that were associated with a significant increase in fatty acid oxidation rate (Figure 10E, * *p* < 0.05).

As shown in Figure S4, feeding mice with a HFD-F resulted in profound alteration of bile acid pool, marked by a severe reduction of Tauro-cholic acid (T-CA). In contrast, the content of other primary bile acids such as Tauro-βmuricholic acid (T-βMCA) were markedly increased in response to HFD-F exposure (Figure S4A). This bile acid pattern suggests an inhibition of FXR signaling, because T-CA is the predominant FXR agonist in mice, and T-βMCA functions as an FXR antagonist [36]. Treating mice with Mt-P did not reverse this pattern, thus revealing that AhR activation resulted in an FXR antagonism (Figure S4A). These data were confirmed also by the analysis of expression of Shp and Cyp7a1 genes (Figure S4B, ^#^ *p* < 0.05 versus naïve mice, * *p* < 0.05 versus HFD-F mice).

As shown in Figures S5 and S6, the beneficial effects exerted by Mt-P on body weight and glucose metabolism and biochemistry were completely abrogated by AhR gene ablation.

## 4. Discussion

Similarly, to other flavonoids, pelargonidins have been used in traditional medicine in the treatment of a number of human alignments [37]. However, because of their poor systemic bioavailability and unpredictable pharmacokinetic, their clinical efficacy remains controversial [20,38,39,40,41,42,43,44].

In the present study, we report the chemical synthesis and pharmacological characterization of a methylated derivative of pelargonidin (Mt-P). By using in vitro and in vivo approaches, we have shown that this agent binds and activates the AhR, a nuclear receptor widely expressed in the intestine, liver, and metabolic tissues. Results of transactivation experiments carried out in AhR-overexpressing HepG2 cells demonstrate that Mt-P transactivates the AhR at an EC_50_ of ~2 µM, and was ~2-fold more potent than the natural compound. The synthetic agent was also significantly more effective than the parent compound in modulating effector functions elicited by exposure of macrophage cell line or freshly isolated macrophages to LPS/IFNγ. In this in vitro setting, in contrast to the natural pelargonidin, Mt-P inhibited *Il-1β*, *Tnfα*, and *Il-6* genes transcription in an AhR-dependent manner. Moreover, while Mt-P significantly increased the production of Tgfβ and Il-10, two signature cytokines for M2-polarized macrophages, the natural compound was only partially effective in increasing the Il-10 gene expression. These regulatory effects, were at least partially due a direct regulation of gene transcription. Indeed, data obtained in vitro in RAW264.7 cells, a macrophage cell line, by a ChIP assay demonstrate that exposure to Mt-P increases the binding of AhR/ARNT dimer on the promoter of its target gene, *Cyp1a1* [45]. Taken together, these data highlight that the Mt-P is a robust AhR ligand in vitro [46], and AhR gene ablation abrogates the anti-inflammatory activities of Mt-P in these settings.

Based on these in vitro findings, we then sought confirmation of whether the Mt-P effectively modulates macrophages and T cell polarization in vivo in a mouse model of intestinal inflammation. The model of colitis induced by intra-rectal administration of TNBS is a Th1-mediated mouse model of colitis that shows similarities with immune dysfunctions found in Crohn’s disease patients. In this model, treating TNBS mice with Mt-P dose-dependently rescued from development of colitis in an AhR-dependent manner. A dose of 5 mg/kg/day of Mt-P fully prevented the development of “clinical” signs and symptoms of colitis, as measured by assessment of CDAI, body weight changes, colon length, and histopathologic changes, and reversed inflammation and immune dysfunction. These effects were AhR-dependent and abrogated by AhR gene ablation. Thus, not only did AhR^−/−^ mice develop a severe disease resulting in a four-day mortality of ~80%, but Mt-P failed to rescue from disease development in these animals. These in vivo beneficial effects were supported by a robust down-regulation of the levels of colon expression of pro-inflammatory cytokines (*Il-1β, Il-6, Tnf-α* and *Ifn-ƴ* mRNA), while increased the levels of *Tgfβ* and *Il-10* genes, two anti-inflammatory cytokines. Consistent with these data, the phenotypic characterization by flow cytometry analysis of immune cells isolated from colonic lamina propria demonstrates that treating colitic mice with Mt-P not only reduced the amount of pro-inflammatory cells, but increased the number of CD4^+^FoxP3^+^ cells, i.e., Treg cells, as well as CD11+GR-IL-10+, i.e., M2 macrophages. Together these findings support the notion that Mt-P activates the AhR in intestinal immune cells, inhibiting the skew toward M1 and Th1 responses by altering the production of pro-inflammatory mediators and promoting the development of M2, tolerogenic, macrophages, and Treg cells. In addition to a direct modulation of intestinal immunity, Mt-P increased the intestinal expression of AhR and its target genes, *Cyp1a1* and *Cyp1b1*, in this model. Furthermore, Mt-P effectively promoted migration and survival of intestinal epithelial cells in vitro, suggesting that AhR activation by Mt-P could contribute to the maintenance of healthy state of the intestinal barrier [47]. 

Because maintenance of intestinal homeostasis is increasingly recognized as an important therapeutic target in obesity and NASH, we then investigated whether Mt-P was beneficial in a rodent model of diet-induced NASH. NASH is a highly prevalent human disorder associated with metabolic syndrome and sub-clinical inflammation, for which therapy remains suboptimal [14,46,47,48,49]. Results shown in Figure 8, Figure 9 and Figure 10 and supplementary Figures S4–S6 demonstrate that treating mice with Mt-P at the dose of 5 mg/kg/day effectively protected from body weight gain, development of insulin resistance, and metabolic dysfunction caused by HFD-F. These beneficial effects were abrogated by AhR gene ablation, supporting the concept that AhR activation by Mt-P was the main mechanism underling the pharmacological effects of the pelargonidin derivative in this model. 

In addition to attenuation of body weight gain, treating mice with Mt-P effectively improved serum biochemistry, and reduced triglycerides, cholesterol plasma levels, and LDL-C, while levels of HDL-C remained unchanged. Analysis of the liver transcriptoma demonstrated that treating mice with Mt-P redirected the expression of several genes involved in lipid metabolism, including Srebp1c and Fasn, a gene that encodes for the fatty acid synthase, a rate-limiting enzyme involved in fatty acid synthesis. Thus, while feeding mice a HFD increased liver lipid biosynthesis, this effect was reversed by AhR activation. However, in contrast to the beneficial effects exerted on plasma biochemistry, treatment of HFD-F mice with Mt-P worsened liver histopathology, resulting in dramatic exacerbation of hepatocytes ballooning. These findings are consistent with a previous study showing that activation of AhR dissociated fatty liver metabolism from insulin resistance by inducing FGF-21. Accordingly, it has been shown that while AhR transgenic mice were protected from HFD–induced obesity and insulin resistance, they developed severe hepato-steatosis. This finding suggests that, at least in mice, systemic AhR activation can dissociate the severity of liver steatosis from insulin sensitivity, and that intake of natural AhR ligand should be carefully evaluated in NASH patients.

Previous studies have demonstrated that AhR activation in the liver induces the expression of FGF-21. It is known that FGF21 exerts multiple metabolic effects, including attenuation of body weight gain and insulin resistance [50]. FGF-21 is almost exclusively produced by the liver and acts on hepatocytes in an endocrine or paracrine fashion by binding to FGF receptor 4, which uses the protein β-klotho as a co-receptor. Because treatment of obese mice with FGF21 leads to weight loss and increased energy expenditure through the activation of eWAT or BAT [51,52], it is likely that FGF-21 mRNA up-regulation in the liver might explain some of the beneficial effects exerted by Mt-P. Importantly, FGF-21 is a direct transcriptional target of AhR, and AhR/ARNT binding sites have been identified in the FGF-21 promoter; although, these AHR-RE overlap binding sequences for peroxisome proliferator-activated receptor α (PPARα), carbohydrate response element-binding protein (ChREBP), and cAMP response element-binding protein hepatocyte specific (CREBH), which also regulate FGF21 gene transcription in hepatocytes [53].

Despite Mt-P-attenuated body weight gain, this agent promoted liver lipid accumulation. Several changes caused by Mt-P might explain this effect. First of all, Mt-P treatment resulted in an AhR-dependent regulation of the expression of Cd36, a gene encoding for the hepatic fatty acid translocase, a membrane transporter involved in the “reverse” cholesterol transport [54]. CD36 binds long-chain fatty acids and facilitates their transport into cells, contributing to the pathogenesis of metabolic disorders such as diabetes and obesity [55]. CD36 is, quantitatively, the most important scavenger receptor for uptake of oxidized lipoproteins by hepatocytes, and previous studies have shown that its upregulation is associated with insulin resistance, hyper-insulinemia, and increased steatosis in patients with NASH. Thus, its up-regulation is consistent with an enhanced FFA uptake by hepatocytes. In addition to an enhanced uptake of circulating lipids by hepatocytes, treating HFD-F mice with Mt-P caused a dramatic down-regulation of Apob100 and ApoC, two genes involved in lipoprotein assembly. Apob100 is the primary liver apolipoprotein and is the carrier for chylomicrons, low-density lipoprotein (LDL), very low-density lipoprotein (VLDL), and intermediate density lipoprotein (IDL), while it is not found in HDL. Because ApoB-100 is necessary for assembly and secretion of VLDL by the liver, and might also serve as the primary ligand for LDL receptor medicated clearance of LDL particles from the blood, our data suggest that accumulation of liver lipids in response to AhR activation might result from a decreased lipid secretion due to an ApoB100-dependent impairment of VLDL formation and secretion by hepatocytes [56]. Liver steatosis deterioration was not due to inflammation, since AhR activation by Mt-P resulted in a robust attenuation in the expression of inflammatory biomarkers in the intestine, liver, and eWAT, confirming that activation of the Mt-P/AhR pathway drives anti-inflammatory signaling. Since HFD-related gut barrier dysfunction is a source of gut, WAT and liver inflammation, and insulin resistance [57], and AhR exerts beneficial effects on the intactness of the gut epithelial barrier [1], attenuation of epithelial barrier dysfunction caused by Mt-P in this model might contribute to the beneficial effects on insulin sensitivity.

In conclusion, we have reported the development of a synthetic derivative of pelargonidin, Mt-P, that activates the AhR and exerts robust anti-inflammatory activity in vitro and in vivo in a AhR-dependent manner. Long-term administration of Mt-P in the setting of mice fed a HFD-F ameliorates insulin sensitivity and attenuates body weight gain, but worsens VLDL biosynthesis and secretion by hepatocytes. The study demonstrates the utility of harnessing nutraceuticals to develop novel pharmacological agents, but also highlights the potential side effects of poorly-investigated nutraceuticals.

## Supplementary Materials

The following are available online at https://www.mdpi.com/2072-6643/11/8/1820/s1. Figure S1: In vitro selectivity of P and Mt-P. Figure S2: P and Mt-P reducing the severity of colitis in a dose dependent manner. Figure S3: Comparison of the beneficial effect of Mt-P and dexamethasone in a mouse model of colitis. Figure S4: Gallbladder bile acids composition. Figure S5: Effects of HFD-F on Ahr^-/-^ mice. Figure S6: Analisys of HFD-F model in hepatic and extrahepatic tissues on Ahr^-/-^ mice.

## Abbreviations

AhR	aryl hydrocarbon receptor
ARNT	AhR nuclear translocator
Hsp90	heat shock protein 90
XREs	xenobiotic response elements
P	pelargonidin
Mt-P	methylated pelargonidin
IBD	inflammatory bowel disease
NASH	non-alcoholic steato-hepatitis
HFD-F	high fat diet and fructose
WAT	white adipose tissue
BAT	brown adipose tissue
eWAT	epididymal adipose tissue
OGTT	oral glucose tolerance test
BMI	body mass index
LDL	low density lipoproteins
HDL	high density lipoproteins
H&E	Hematoxilin and Eosin
T-CA	Tauro-cholic acid
T-βMCA	Tauro-βmuricholic acid
