# Supplementary material for: The Aryl Hydrocarbon Receptor (AhR) Mediates the Counter-Regulatory Effects of Pelargonidins in Models of Inflammation and Metabolic Dysfunctions
Source: Nutrients. 2019 Aug 7;11(8):1820. doi: 10.3390/nu11081820 (PMC6723439; doi:10.3390/nu11081820)
Supplement: Supplementary file 1 [file nutrients-11-01820-s001.pdf]

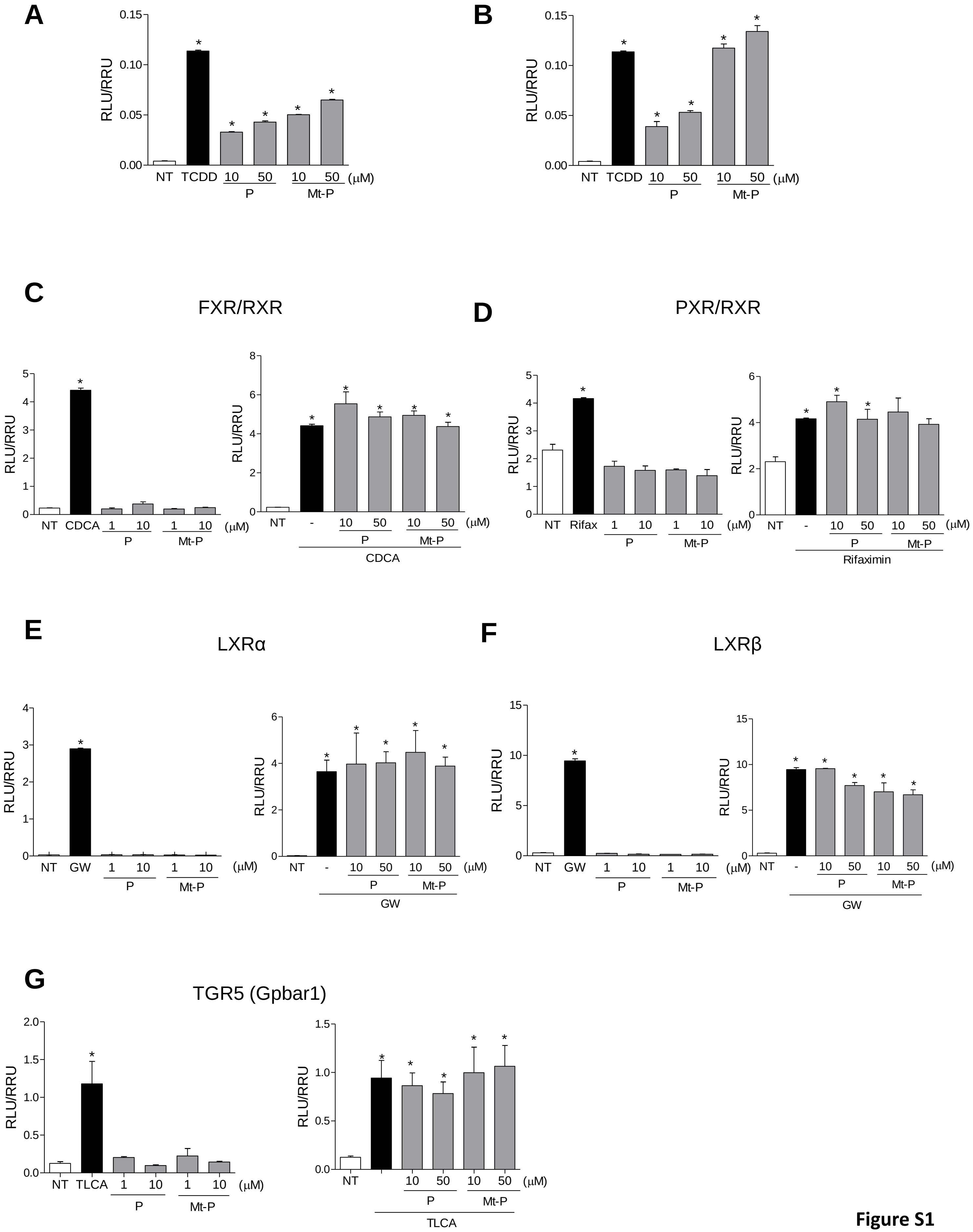
A
B
RLU/RRU
0.00
0.05
0.10
0.15
NT
TCDD
10
50
(μM)
P
Mt-P
C
FXR/RXR
CDCA
D
PXR/RXR
Rifax
Rifaximin
E
LXRα
GW
F
LXRβ
G
TGR5 (Gpbar1)
TLCA
-
1
10

Figure S1

Figure S1. HepG2 cells were transiently transfected with 200 ng of reporter vector pLIghtAhRE, 100 ng of pCMVXL4AhR and 100 ng of pGL4.70 Renilla. 24 hours post-transfection cells were stimulated with two concentrations of (**A**) normal or (**B**) acidified P and Mt-P to evaluate AhR agonism (10 and 50 μM). Panels **C-F**: Specificity of P and Mt-P on other nuclear receptors**.** (**C**) HepG2 cells were co-transfected with pSG5-FXR, pSG5-RXR, and with the reporter vector phsp27TKLuc, or (**D**) with 100 ng pSG5-PXR, 100 ng pSG5-RXR, 100 ng pGL4.70 Renilla and 200 ng reporter vector containing the PXR target gene promoter (CYP3A4 gene promoter) cloned upstream of the luciferase gene (pCYP3A4promoter-TKLuc) or (**E-F**) with the Gal4 luciferase reporter vector and with a chimera in which the Gal4 DNA binding domain is fused to the LBD of LXRα or LXRβ; (**G**) HEK-293T cells were co-transfected with GPBAR1 and a reporter gene containing a cAMP responsive element in front of the luciferase gene. At 24 h post-transfection, cells were stimulated 18 h with 10 μM CDCA, 500 nM Rifaximin, 10 μM GW3965 or 10 μM TLCA and both P. and Mt-P (10 and 50 μM) alone or in combination with the different control agonist. Value was expressed as RLU/RRU (Relative Luciferase Unit/Relative Renilla Unit). Results are expressed as mean ± standard error. (n=3) *$p<0.05$ versus not treated cells (NT).

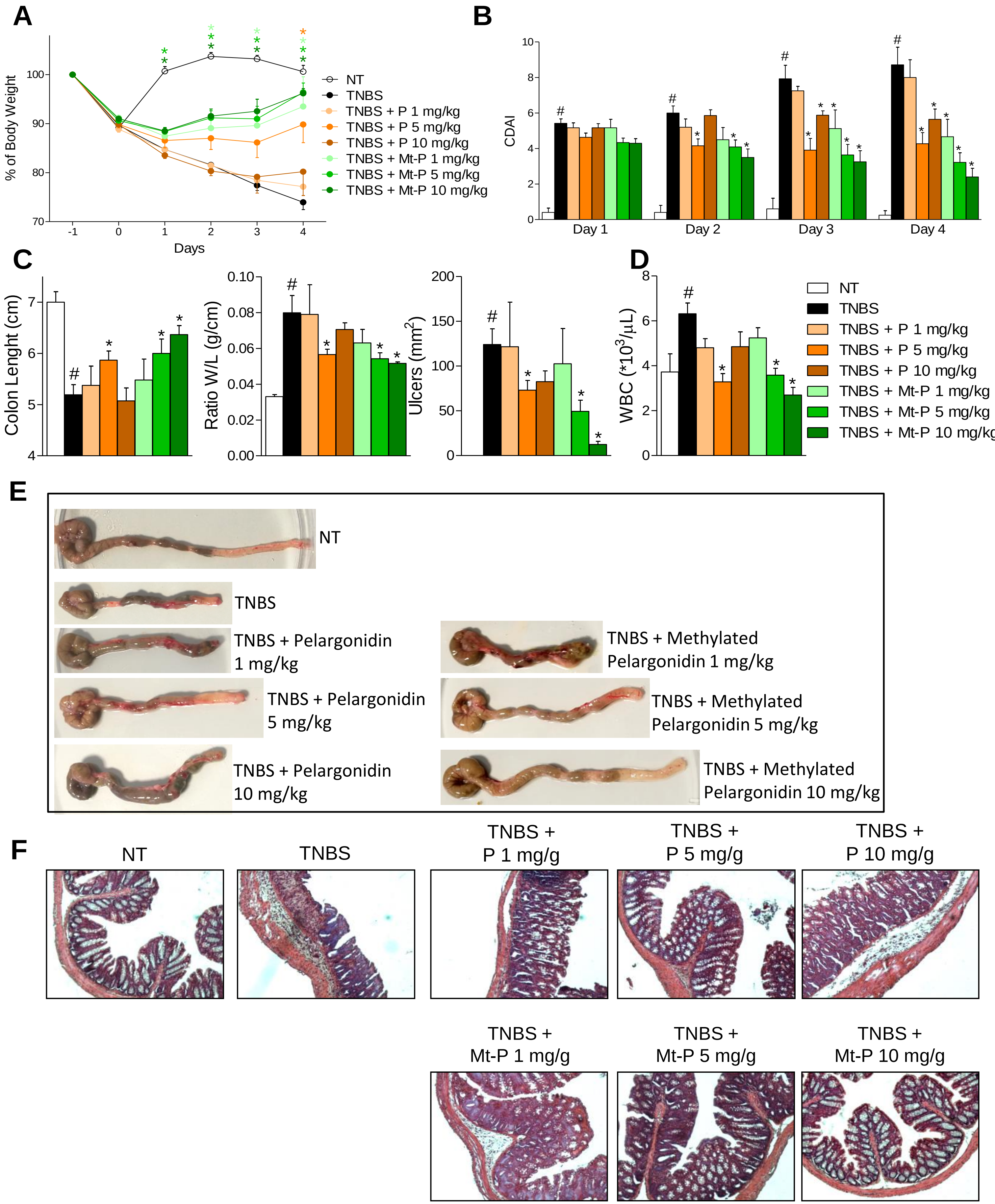
A
% of Body Weight
Days
NT
TNBS
TNBS + P 1 mg/kg
TNBS + P 5 mg/kg
TNBS + P 10 mg/kg
TNBS + Mt-P 1 mg/kg
TNBS + Mt-P 5 mg/kg
TNBS + Mt-P 10 mg/kg
B
CDAI
Day 1
Day 2
Day 3
Day 4
C
Colon Lenght (cm)
Ratio W/L (g/cm)
Ulcers (mm2)
D
WBC (*10³/μL)
E
NT
TNBS
TNBS + Pelargonidin 1 mg/kg
TNBS + Methylated Pelargonidin 1 mg/kg
TNBS + Pelargonidin 5 mg/kg
TNBS + Methylated Pelargonidin 5 mg/kg
TNBS + Pelargonidin 10 mg/kg
TNBS + Methylated Pelargonidin 10 mg/kg
F
NT
TNBS
TNBS + P 1 mg/g
TNBS + P 5 mg/g
TNBS + P 10 mg/g
TNBS + Mt-P 1 mg/g
TNBS + Mt-P 5 mg/g
TNBS + Mt-P 10 mg/g

Figure S2

Figure S2. Balb/C mice were treated with TNBS alone or in combination with P or Mt-P (1, 5 or 10 mg/kg) as described before (see Material and Methods). Changes in (**A**) body weight and (**B**) CDAI of mice during the course of TNBS-induced colitis. (**C**) Analysis of the intestinal inflammatory score: colon length (left), ratio of colon weight/colon length (central) and ulcers (right). (**D**) Number of with blood cells (WBCs) at the day 4. (**E**) Photographs of colon and (**F**) H&E staining of colon from control mice and from mice treated with TNBS or TNBS plus various concentrations of two compounds. Values are normalized relative to Gapdh mRNA and are expressed as mean ± SEM of six/twelve mice per group (NT=6; TNBS=10; TNBS + P 1 mg/kg= 6; TNBS + P 5 mg/kg=8; TNBS + P 10 mg/kg=10; TNBS + Mt-P 1 mg/kg=8; TNBS + Mt-P 5 mg/kg=12; TNBS + Mt-P 10 mg/kg=10) . (p#<0.05 vs NT mice, p*<0.05 vs TNBS mice).

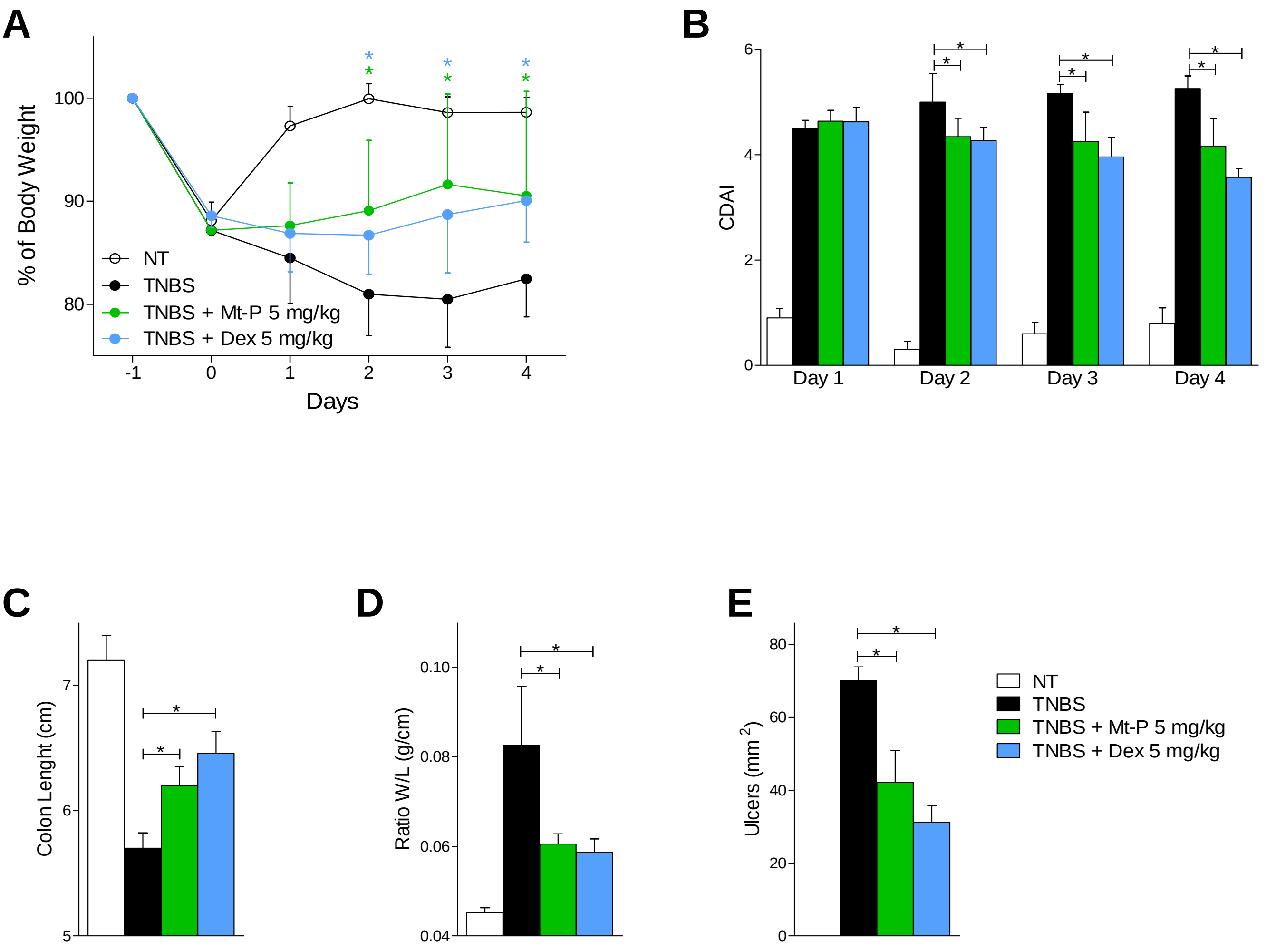

Figure S3. Balb/c mice were treated with TNBS and then administered with vehicle or Mt-P 5mg/kg/day or dexamethasone 5 mg/kg/day. Changes in (**A**) body weight and (**B**) CDAI score. Analysis of the intestinal inflammatory score: (**C**) colon length, (**D**) ratio of colon weight/colon length and (**E**) ulcers. Values are normalized relative to Gapdh mRNA and are expressed as mean ± SEM of five/ten mice per group (NT=5; TNBS=8; TNBS + Mt-P 5 mg/kg=10; TNBS + Dex 5 mg/kg=10). (p*<0.05).

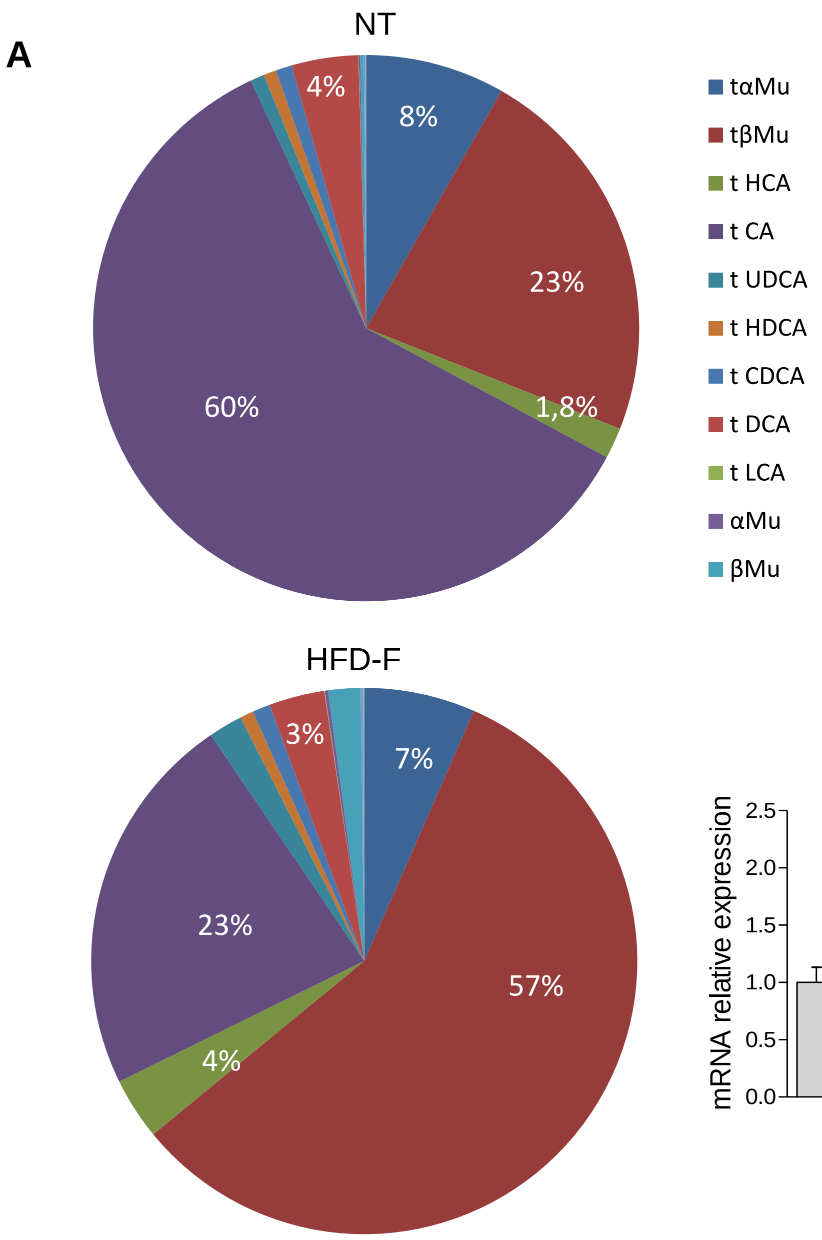

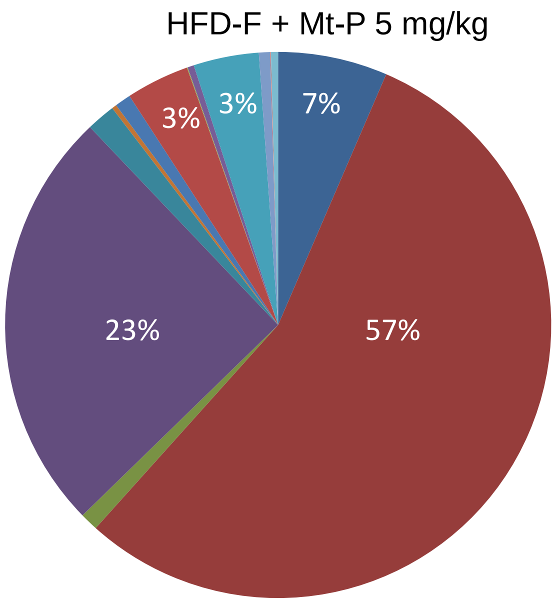

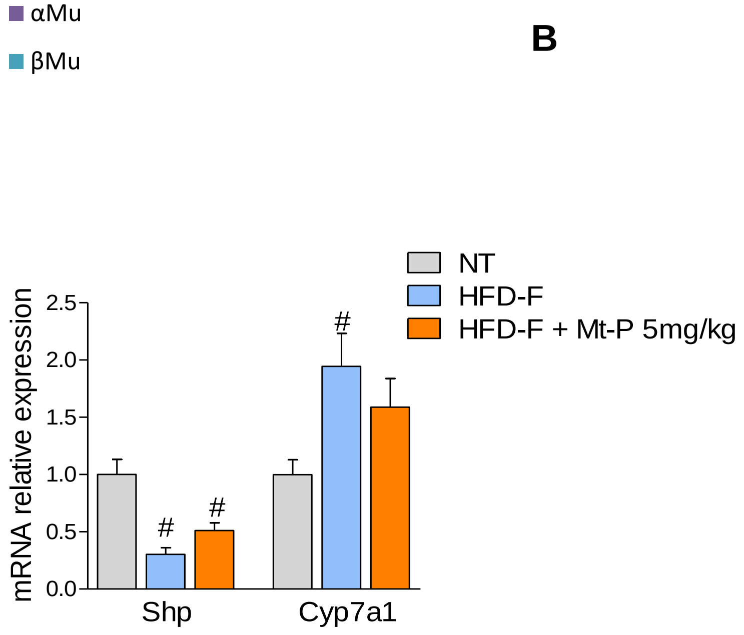

Figure S4. C57BL/6 male mice were fed a high fat diet (HFD) and fructose or normal chow diet as described before (see Material and Methods). (**A**) Percentage composition of gallbladder bile acids pool. (**B**) Relative mRNA expression of Shp and Cyp7a1 in liver. Values are normalized relative to Gapdh mRNA and are expressed as mean ± SEM of six/ten mice per group (NT=6; HFD-F=8; HFD-F + Mt-P 5 mg/kg=10). (p#<0.05 vs NT, p*<0.05 vs HFD-F).

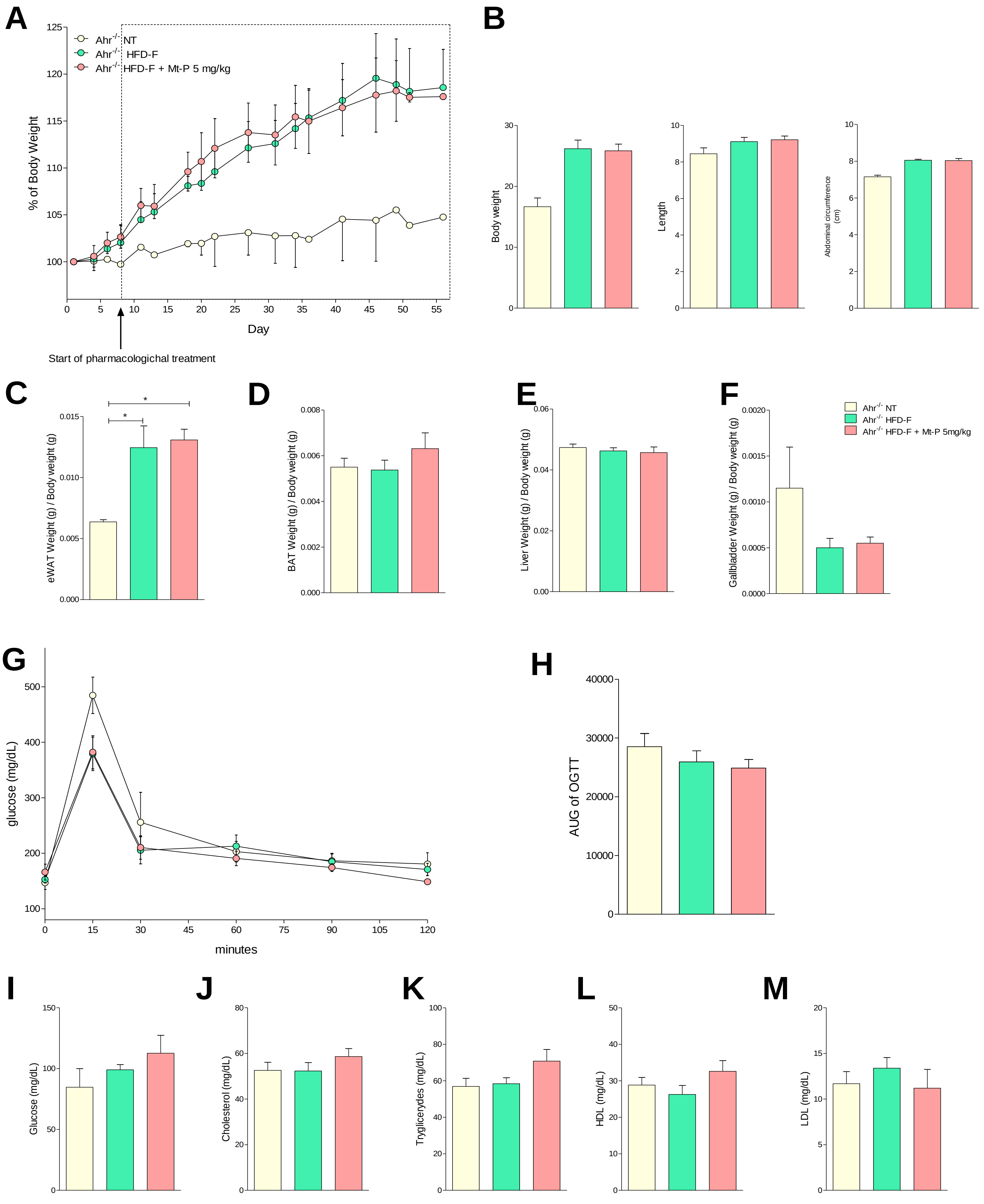
A
Ahr-/- NT
Ahr-/- HFD-F
Ahr-/- HFD-F + Mt-P 5 mg/kg
% of Body Weight
Day
Start of pharmacologichal treatment
B
Body weight
Length
Abdominal circumference (cm)
C
eWAT Weight (g) / Body weight (g)
D
BAT Weight (g) / Body weight (g)
E
Liver Weight (g) / Body weight (g)
F
Gallbladder Weight (g) / Body weight (g)
Ahr-/- NT
Ahr-/- HFD-F
Ahr-/- HFD-F + Mt-P 5mg/kg
G
glucose (mg/dL)
minutes
H
AUG of OGTT
I
Glucose (mg/dL)
J
Cholesterol (mg/dL)
K
Tryglicerydes (mg/dL)
L
HDL (mg/dL)
M
LDL (mg/dL)

Figure S5

Figure S5. Ahr$^{-/-}$ male mice were fed a high fat diet (HFD) and fructose or normal chow diet as described before (see Material and Methods). (**A**) Changes in body weight (%) assessed weekly. Anthropometrical parameters: (**B**) BMI measured at the end of the study. Ratio (**C**) eWAT weight/body weight, (**D**) BAT weight/body weight, (**E**) liver weight/body weight, (**F**) gallbladder weight/body weight. (**G**) Glucose plasma levels in response to OGTT; (**H**) AUCs of glucose plasma levels expressed in arbitrary units. Serum levels of (**I**) Glucose, (**J**) Cholesterol, (**K**) Triglycerides, (**L**) HDL and (**M**) LDL. Biochemistry values were measured at the end of the study. The data are mean ± SEM of six/ten mice per group (Ahr$^{-/-}$ NT=6; Ahr$^{-/-}$ HFD-F=10; Ahr$^{-/-}$ HFD-F + Mt-P 5 mg/kg=10). (p*<0.05).

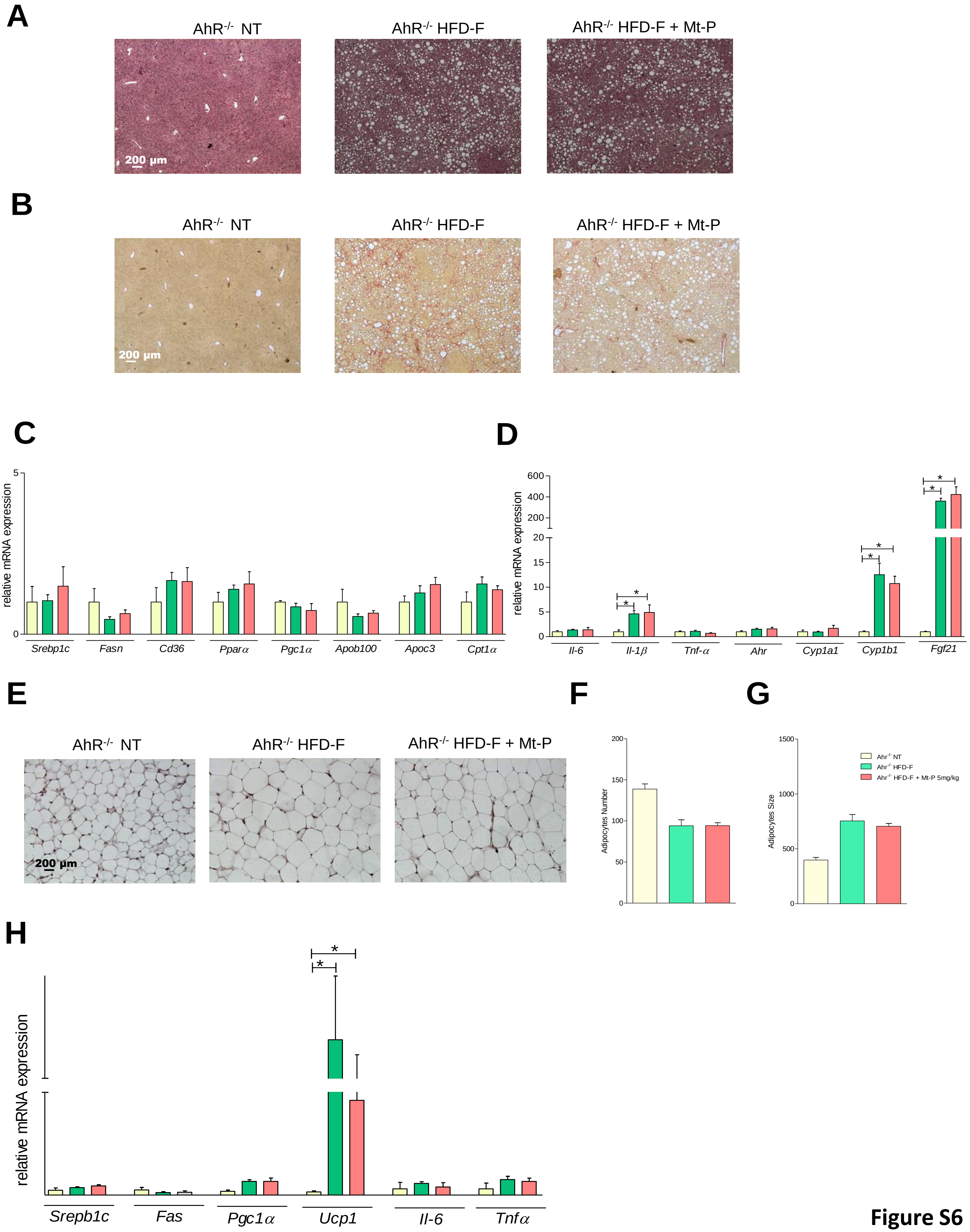

A
AhR-/- NT
AhR-/- HFD-F
AhR-/- HFD-F + Mt-P
200 µm
B
AhR-/- NT
AhR-/- HFD-F
AhR-/- HFD-F + Mt-P
200 µm
C
relative mRNA expression
5
0
Srebp1c
Fasn
Cd36
Pparα
Pgc1α
Apob100
Apoc3
Cpt1α
D
relative mRNA expression
600
400
200
20
15
10
5
0
*
Il-6
Il-1β
Tnf-α
Ahr
Cyp1a1
Cyp1b1
Fgf21
E
AhR-/- NT
AhR-/- HFD-F
AhR-/- HFD-F + Mt-P
200 µm
F
Adipocytes Number
200
150
100
50
0
G
Adipocytes Size
1500
1000
500
0
Ahr-/- NT
Ahr-/- HFD-F
Ahr-/- HFD-F + Mt-P 5mg/kg
H
relative mRNA expression
*
Srepb1c
Fas
Pgc1α
Ucp1
Il-6
Tnfα

Figure S6

Figure S6. Ahr$^{-/-}$ male mice were fed a high fat diet (HFD) and fructose or normal chow diet as described before (see Material and Methods). (**A**) Hematoxylin and eosin (H&E) staining of liver tissues obtained at the end of the study. (Magnification 4x). (**B**) Sirius Red staining of liver tissues obtained at the end of the study. (Magnification 10x). The images shown in panels B-D are representative of at least 10 others, each one obtained from an individual mouse, showing a similar pattern of regulation. Hepatic expression of (**C**) genes involved in the regulation of lipid synthesis (Srebp1c, Fas), fatty acids oxidation (Pparα, Pgc1α, Cpt1α) and VLDL formation (Apo100B, ApoC), and (**D**) pro-inflammatory genes (Il-6, Tnfα, Il-1β, F4/80), and Ahr-target genes (Ahr, Cyp1a1, Cyp1b1, Fgf21). (**E**) Hematoxylin and eosin (H&E) staining of white adipose tissue sections obtained at the end of the study. The images shown are representative of at least 10 others, each one obtained from an individual mouse, showing a similar pattern of regulation. Magnification 4x. (**F**) Adipocytes number and size obtained from analysis of H&E staining performed with Image J. (**G**) Changes in mRNA expression of eWAT genes. (**H**) Changes in mRNA expression of eWAT genes. Values are normalized relative to Gapdh mRNA and are expressed as mean ± SEM of six/ten mice per group group (Ahr$^{-/-}$ NT=6; Ahr$^{-/-}$ HFD-F=10; Ahr$^{-/-}$ HFD-F + Mt-P 5 mg/kg=10). ($p^* < 0.05$).
